# The Overview of Porous, Bioactive Scaffolds as Instructive Biomaterials for Tissue Regeneration and Their Clinical Translation

**DOI:** 10.3390/pharmaceutics12070602

**Published:** 2020-06-29

**Authors:** Gaëtan Lutzweiler, Albana Ndreu Halili, Nihal Engin Vrana

**Affiliations:** 1 Institut National de la Santé et de la Recherche Medicale, UMR_S 1121, 11 rue Humann, 67085 Strasbourg CEDEX, France; 2 Department of Information Technology, Aleksander Moisiu University, 2001 Durres, Albania; albanahalili@uamd.edu.al; 3 Spartha Medical, 14B rue de la Canardière, 67100 Strasbourg, France

**Keywords:** porous scaffolds, pore size, tissue engineering, bioactive agent delivery, interconnection size

## Abstract

Porous scaffolds have been employed for decades in the biomedical field where researchers have been seeking to produce an environment which could approach one of the extracellular matrixes supporting cells in natural tissues. Such three-dimensional systems offer many degrees of freedom to modulate cell activity, ranging from the chemistry of the structure and the architectural properties such as the porosity, the pore, and interconnection size. All these features can be exploited synergistically to tailor the cell–material interactions, and further, the tissue growth within the voids of the scaffold. Herein, an overview of the materials employed to generate porous scaffolds as well as the various techniques that are used to process them is supplied. Furthermore, scaffold parameters which modulate cell behavior are identified under distinct aspects: the architecture of inert scaffolds (i.e., pore and interconnection size, porosity, mechanical properties, etc.) alone on cell functions followed by comparison with bioactive scaffolds to grasp the most relevant features driving tissue regeneration. Finally, *in vivo* outcomes are highlighted comparing the accordance between *in vitro* and *in vivo* results in order to tackle the future translational challenges in tissue repair and regeneration.

## 1. Introduction

The first concepts of tissue engineering (TE) were proposed by Langer et al. [1] where the idea was to expand cells of a given patient *in vitro* prior seeding them into a synthetic or natural scaffold followed by the implantation of this cellularized scaffold back to the patient. TE appears as a promising way to regenerate a part of the body which has been lost or damaged after trauma or diseases, especially for large defects and for poorly vascularized tissues having limited healing capacities such as cartilage. Many researchers used to generate porous scaffolds for tissue regeneration where the scaffold acts as an extracellular matrix (ECM) substitute for cells allowing them to adhere, proliferate, and to differentiate while the scaffold is gradually degraded and replaced by a new tissue. This is mainly based on the fact that many types of materials appeared to be biocompatible. Even though the definition of biocompatibility is still unclear depending on whether studies are conducted *in vitro* or *in vivo*, it is generally accepted that a material is considered as biocompatible as long as it does not promote any acute toxicity or alteration of cell functions [2]. Furthermore, a large number of ways to form these scaffolds already exist (foaming, electrospinning, salt leaching, additive manufacturing, self-assembly, etc.), offering many options to tailor the structure of the scaffold according to the targeted application. Tissue engineering’s market is estimated to reach USD 11.5 million in 2022 [3].

Scaffolds offer a three-dimensional environment to cells which was already shown to modify the genetic expression and shape of cells compared to flat surfaces [4] and the architecture of scaffolds can influence cell fate such as their proliferation or their ability to colonize the structure. Moreover, the substrate chemistry or topography impacts the adhesiveness, and later on, the proliferation of cells and the addition of soluble molecules such as growth factors or hormones contributes as well to the overall cellular response [5,6]. However it is not straightforward to determine whether cell response into a three-dimensional structure is mainly driven by the cell–substrate interaction or by the macro structure of the scaffold since cells can probe their environment at different levels ranging from the nanoscale such as protein conformation with which they interact within the ECM and up to the macroscale corresponding to the global tissue organization [7,8]. Therefore, the main parameters which can lead to full scaffold integration and sustain cell functions remain unclear. Consequently, this review firstly summarizes the main materials used in TE and their techniques of fabrication. Thereafter, focusing on the interaction between the scaffold architecture solely namely: pore and interconnection sizes, porosity, and mechanical properties and the associated cell response before reviewing the impact of bioactive scaffolds where biological cues are supplemented to the scaffold. The idea is to investigate if structural parameters can be enough to control tissue growth compared to bioactive scaffolds providing signaling molecules and other cues interacting with cells. Numerous studies were already conducted, trying several conditions to promote tissue growth emanating various conclusions. This review aims to evaluate the degree of complexity required to generate an ideal scaffold gathering the most relevant parameters that drive tissue regeneration. Finally, the main feedbacks of *in vivo* studies will be provided to highlight the main successes while emphasizing future challenges to continue to improve the integration of implantable scaffolds in the body.

## 2. Overview of the Techniques Used for Fabrication of Porous Scaffolds for Tissue Engineering Applications

Scaffolds have been generated by numerous techniques offering various degrees of flexibility according to the final application (i.e., type of cell hosted, soft or hard tissues, load-bearing locations or not). Ideally, the obtained scaffold must provide a well-balanced environment between mechanical properties, high specific surface area, good diffusivity and being well interconnected to support and sustain cell functions. An overview of the most frequently used techniques of preparation and the resulting scaffolds will be given here along with their many advantages and limitations for tissue engineering applications.

### 2.1. Electrospinning

**Figure 1:**
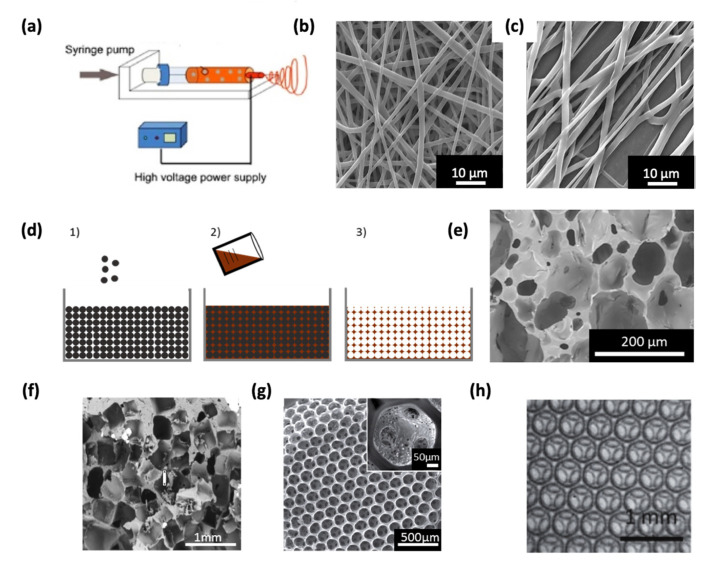
Illustration of the electrospinning process (**a**). Taken with permission from Li et al. [55] SEM images showing the fiber assembly either randomized (**b**) or aligned (**c**), adapted with permission from Ndreu et al. [11]. (**d**) Illustration of particle leaching process with the template formation (1) followed by matrix pouring (2) and particle dissolution (3) taken from Lutzweiler [56], SEM images of scaffolds generated by chemical foaming (**e**) taken with permission from Ng et al. [57], particle leaching using salt (**f**) taken with permission from Janik et al. [58], sphere templating (**g**) taken with permission from Choi et al. [59], and microfluidics (**h**) reused with permission from Testouri et al. [48].

Electrospun assemblies are obtained by applying an electric field to a charged polymer solution which is propelled to a static or non-static collector either oppositely charged or grounded [9]. Highly fibrous structures can therefore be obtained having similarities with the native ECM (Figure 1a,b). The fiber morphology and size can be controlled through various parameters such as polymer type and solution concentration, voltage applied, collector-to-needle tip distance, and conductivity of the polymeric solution [10,11] (Figure 1c). Polymers, both natural and synthetic, are widely employed in electrospinning due to their relatively low melting temperature and ease to process [12]. Additionally, electrospun scaffolds can be functionalized in order to incorporate relevant properties such as biodegradability or drug release [13,14,15]. Furthermore, the control over mechanical properties of the fibrous structure can also be achieved [16,17,18]. However, even though a tight packing of the fibers allows to maintain an acceptable mechanical strength, the high fiber density leads to structures with small pores which consequently limits the cellular infiltration throughout the overall scaffold [19]. Another consequence that follows the previous statement is the reduction of the transport properties across the whole volume, hampering an efficient supply of nutrients and oxygen to cells [20]. Thereby, electrospinning appears as a biomimetic approach to produce ECM-like assemblies with high surface-to-volume ratios, but the balance between mechanical properties and porosity needs to be adjusted to guarantee an efficient colonization of cells. Interestingly, Kuo et al. [21] recently developed a co-electrospinning system where fibers of two types were intertwined. After selective dissolution of one type of fiber, the interstitial space was increased, showing beneficial effects on cell growth.

### 2.2. Additive Manufacturing

Additive manufacturing, which includes 3D printing, selective laser sintering (SLS) or stereolithography, among others [22], groups a set of techniques where some material is added during the process conversely to etching whose process relies on material removal during the process. Additive manufacturing is standardized by the American Society for Testing and Materials (ASTM) F2792. This technique allows for the rational design of porous materials where the interconnection sizes, pore shapes, and the porosity can be controlled independently, which represents a major advantage for scaffold engineering [23]. Moreover, this technology can be employed for numerous types of materials ranging from ceramics, polymers, metals and composites [24]. Therefore, scaffolds can be designed for a broad range of applications such as tendons [25] muscles [26], bones [27,28], and cartilage [29]. Furthermore, direct printing of cells jointly with the scaffold shows promising results, especially regarding the homogenous distribution of cells within the printed scaffold that is achieved [30,31]. One major drawback of additive manufacturing techniques is the lack of control over the nano up to the macro scale of the final scaffold, which is important considering the multiscale interaction of cells with the surrounding tissues [32].

### 2.3. Particles Leaching

Scaffolds obtained by particle leaching are based on an assembly of particles acting as a negative template. Afterwards, the liquid (generally a monomer, a polymer melt, or solution) is poured on the template and let to infiltrate and to solidify followed by the selective dissolution of the particle leaving a porous network (Figure 1d). The final structure is an accurate reproduction of the initial template whose pore size and shape are fixed by the size and shape of the sacrificial particles which are typically salt [33] (Figure 1f) due to its high solubility in aqueous media and preventing the use of organic solvents. However, other sacrificial agents can be found in the literature, as for example, polymers [34]. This simple approach provides the control on the pore size by fixing the size of the sacrificial agent which can be selected according to the needs, which are usually ranging from tens to hundreds of microns. Additionally, the size of the interconnections can also be controlled through merging of particles packed in the initial template. Different strategies were followed as the addition of an adhesive [35], sintering [36,37], or chemical dissolution [38]. In all cases, the formation of a “neck” between adjacent particles leads to the formation of an interconnection in the porous material. Furthermore, the morphology of the final structure depends only on the morphology of the initial template, hence, a vast number of polymers can be used in this process. In one of our previous studies [37], porous scaffolds where interconnection sizes were controlled in a predictive manner using a new theoretical model of sintering were generated. Nearly identical sacrificial agents such as microspheres can lead to highly homogenous structures (see Figure 1g) which is more likely to be used to investigate the influence of the scaffold’s structure on cell response [38,39,40]. Thus, particle leaching is a simple and cheap method that can be used to generate scaffolds with controlled pore size, porosity, and interconnectivity as well. Additionally, the interconnectivity (i.e., the degree of interconnection) can be improved by using spherical particles where the jamming transition ensures the highest number of interconnection per single pore [41].

### 2.4. Foaming

The use of foams for tissue engineering applications is well-documented in the literature [42]. Foams are mostly obtained from a liquid/viscoelastic matrix, whose solidification leads to the formation of gas bubbles as a side product. These bubbles are then trapped within the solidified matrix which forms a foam. This process is widely used in industry but is also used to fabricate scaffolds for tissue engineering [43,44]. However, the control of architectural parameters including the pore size distribution, the porosity, and the interconnection size remain challenging, thus, the geometry of these scaffolds is rather random as shown in Figure 1e. Conversely, a precise control of the pore size within the micrometric range can be reached through physical foaming and especially by microfluidics. In that case, bubbles are generated one by one in a chip and packed in a container where the bubble assembly forms an initially liquid foam which becomes solid after polymerization [45,46,47]. One advantage of this individualized bubble formation is that the resulting foams have a narrow pore size distribution, therefore the final scaffolds are highly homogenous and reproducible. One can note the similar appearance between scaffolds generated via monodisperse sphere templating and those obtained through microfluidics (Figure 1h vs. Figure 1g). However, the absence of friction between bubbles formed by microfluidics (compared to solid spheres) allows to obtain highly crystalline structures displaying hexagonally close-packed arrangements [48,49] (Figure 1h). In crystalline structures, the coordination number (i.e., the number of direct neighbors) is maximized and the resulting interconnectivity is therefore enhanced. The interconnectivity is likely to be related to mass transport properties and cell migration ability, implying that this parameter needs to be optimized [50]. A few examples describing the formation of scaffolds through microfluidics are currently available. Costantini et al. [51,52] confirmed that alginate scaffolds generated by microfluidics with monodisperse pore size distribution have an improved interconnectivity, and consequently, a better nutrient supply to cells compared to scaffolds prepared by chemical foaming. Furthermore, Dehli et al. [45] generated a gelatin methacryloyl hydrogel-based foam using microfluidics bubbling to produce a macroporous and ordered hydrogel as a promising candidate as a scaffold for tissue engineering. Nevertheless, microfluidics bubbling is still at the early stages of development and many aspects need to be addressed in order to broaden its applications. Indeed, the stability of the initially liquid foam and the solidification time must be finely tuned to ensure an open cell foam. Moreover, the mechanism of pore opening (i.e., interconnection formation) is still unclear, thereby, the distinct control over pore and interconnection size cannot be guaranteed yet [53,54].

### 2.5. Hydrogels

**Figure 2:**
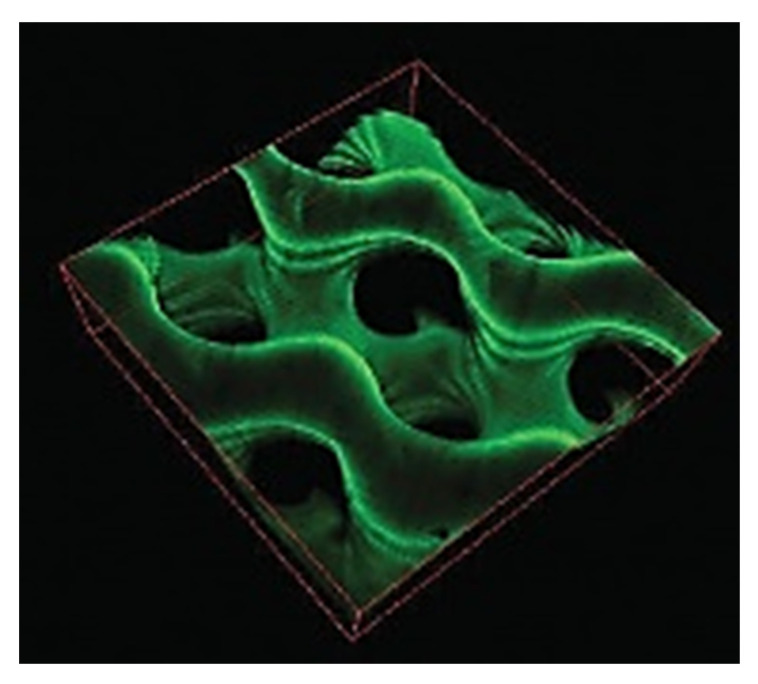
Carboxybetaine methacrylamide (CBMAA)-based porous hydrogel having a gyroid structure. The structure is visualized using a confocal microscope where FITC-BSA is adsorbed on the surface. Adapted with permission from Kostina et al. [79].

Hydrogels are scaffolds whose structure forms a fibrous and highly hydrated structure. They can be used as cell carriers where cells are directly embedded in the fibrous network. Micro-sized meshes can directly be assimilated as an ECM protein network offering a realistic support for cell growth. Hydrogels can be easily obtained by crosslinking a monomer solution either chemically [60], under radiation [61], but also by their self-assembling mechanisms [62] or through an aggregation mechanism after protein denaturation [63]. One can, for instance, find hydrogels based on alginate [64], gelatin [65], hydroxypropylmethyl cellulose [66], or hybrid combinations of fibrinogen and polyethylene glycol [67]. Hydrogel-based scaffolds can be generated by conventional technologies such as 3D printing [68] and also can be coupled to a stiffer scaffold with superior mechanical stiffness [69]. Moreover, supramolecular hydrogels such as self-assembled peptides (SAP)-based hydrogels can be formed enzymatically [70] or spontaneously depending on the initial peptide sequence chosen [71] whilst viscoelasticity can be introduced through non-covalent bonds [72]. Recently, a hydrogel with high mechanical properties a non-swelling behavior that allowed cell proliferation was obtained from crosslinking of micelles at high density [73]. Mechanical properties of hydrogels are also straightforward to handle, through initial monomer concentration [74] or via the crosslinking density [75]. Nevertheless, one major drawback associated with hydrogels is the limited diffusion of nutrients and oxygen throughout the dense fibrous network [76,77]. Such limitations can be overcome by the insertion of macropores or channels in the hydrogel structure [45,78], thereby, one can take advantage of the improved transport properties through macropores combined with the local mesh-like environment mimicking the ECM. Kostina et al. [79] recently printed a porous gyroid structured hydrogel, with a high specific surface area and highly permissive properties for nutrient diffusion as well (Figure 2). However, mechanical properties of hydrogels are sometimes poor due to their highly hydrated nature especially when utilized for hard tissue applications [80]. Another strategy to improve the supply of nutrients is the patterning of the hydrogel with factors involved in the vascularization process to sustain the nutrient supply for longer periods [81]. Nano/micro hydrogel particles, also named as “nano/microgels”, represent an alternative way to work on diffusion problems by increasing the surface-to-volume ratio. Nano/microgels are suitable either for cell or drug delivery systems which can be injected directly by a syringe allowing minimal invasion [82,83].

## 3. Materials Used for Scaffold Compositions

### 3.1. Polymers

Synthetic polymers are widely used in TE and for medical devices since they can be produced in large quantities and are easy to process with low costs. Besides this, the structure–property relationship of polymers is well-established [84]. Biodegradable polymers are intensively studied since scaffolds are expected to be eliminated from the body in time by allowing new tissue replacement. Among these, polyesters (R-COO-R’) are interesting candidates since they are subject to hydrolysis [85]. Polymer degradability is also related to other features such as crystallinity and molecular weight [86]. Common examples which can be found in the literature are poly(methyl methacrylate) (PMMA), poly(lactic-co-glycolic acid) (PLGA), poly(ɛ-caprolactone) (PCL), polylactic acid (PLA), or poly(ethylene terephthalate) (PET) based scaffolds. According to the aim of application, implanted scaffolds are intended for long-term standing. In that case, polyethers (R-O-R’) are more efficient to resist against hydrolysis and polytetrafluoroethylene (PTFE) poly(ether)urethane (PU), or poly(ethylene) glycol (PEG) are options. Nonetheless, implanted devices are subject to harsh conditions within the body and other degradation mechanisms occur, especially enzymatic-mediated ones. Vascular grafts are usually made with PTFE (Teflon^®^), whose fluorocarbon backbone is highly stable and antithrombotic [87]. Moreover, peptides and proteins are polyamides which can also be found in nylon for example. Such materials own advantageous mechanical properties and flexibility due to their intra-molecular hydrogen bonding [88]. Moreover, the surface of polymers can be functionalized by chemical and physical treatments to increase their biological activity [89,90,91,92].

**Figure 3:**
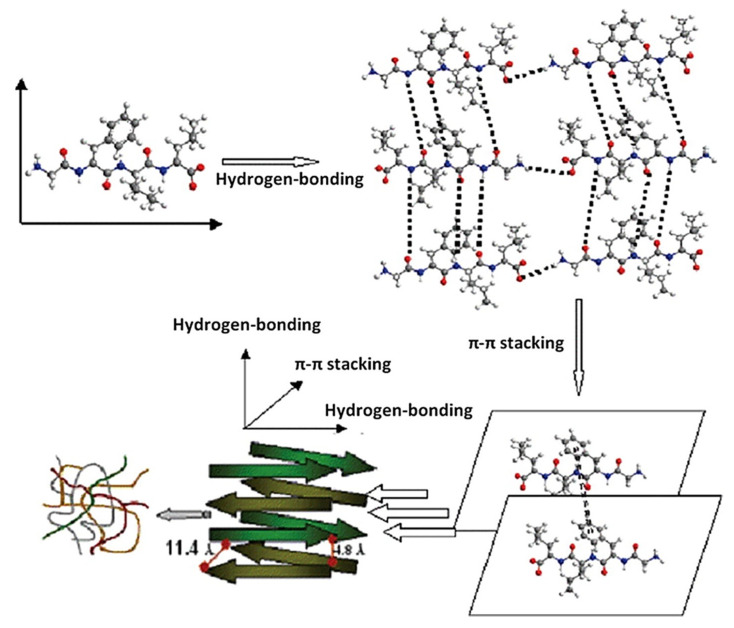
Proposed arrangement of self-assembled peptides (SAP)-based hydrogel from the tetrapeptide (H2N-Gly-Phe-Ile-Leu-COOH) where weak interactions as hydrogen bonding and π-π interactions govern the cross-*β* structure. Reprinted with permission from Naskar et al. [107]. Copyrights (2020) American Chemical Society.

Polymers derived from natural sources, particularly those derived from extracellular matrix (ECM) are relevant materials for tissue engineering due to their intrinsic bioactivity along with their self-assembling ability [93,94]. For instance, the conformation of fibronectin can orchestrate precisely the delivery of growth factors to cells as shown by Trujillo et al. [95]. Therefore, natural polymers are biocompatible and can support and direct cellular functions unlike synthetic polymers, whose bioactivity often requires surface modifications. Fibrin, collagen, or polysaccharides are frequently used since they are biodegradable and mechanically stable [96,97,98]. Moreover, additional functionalities can be grafted to peptides or protein fragments through amine and carboxylic groups. Hydrogels can be formed from naturally-derived polymers, which provide high water or biological fluids retention [99,100,101]. One major drawback of natural polymers comes from the batch-to-batch variability, responsible for changes in chemical nature or in mechanical properties, rendering such systems difficult to process at large scales [102]. Finally, supramolecular polymers based on reversible and non-covalent chemical bonds [103] are emerging and provide unique properties to materials [104]. For instance, peptides can spontaneously self-assemble in water via hydrogen bonding or π-π interactions leading to a three-dimensional network of self-assembled peptides (SAP). Accordingly, supramolecular chemistry provides a new route to generate SAP-based hydrogels whose benefits were already noticed for cell encapsulation and tissue engineering [105,106]. Naskar et al. [107] used two synthetic tetrapeptides, namely Gly-Phe-Ile-Leu and Gly-Ala-Ile-Leu, and defined how such oligopeptides self-assembled under pH variations (Figure 3).

### 3.2. Ceramics

Another class of materials used in tissue engineering are ceramics, which are inorganic composites that are usually brittle and hard, and they are mostly suitable for hard tissue applications [108]. Based on their properties, they are categorized in three different classes, namely: bioinert, bioactive, and bioresorbable. Bioinert ceramics are commonly used at locations requiring high load-bearing abilities such as articulations. They have a good stability *in vivo* and therefore sustain their mechanical properties for long-term. These materials are widely employed for orthopedics [109]. Aluminum oxide, zirconium oxide, and pyrolytic carbon are common examples. Bioactive ceramics can interact with their physiological environment by creating bonds with bones [110], resulting in a better integration of implants [111]. These ceramics can also be used as coatings of another based-material and are already commercialized, as for instance, Bioglass^®^ [112]. The third class concerns bioresorbable ceramics, which can be dissolved under physiological conditions, or through cell-induced degradation mechanisms [113]. The products of the dissolving reaction are non-toxic and the degradation rate is equivalent to the grow rate of the neo-tissue [114]. Calcium phosphate is the most abundant type of bioresorbable ceramics including hydroxyapatite (Hap), which can be found in teeth and bones, and β-tricalcium phosphate (TCP) is also deeply investigated [115].

### 3.3. Metals

Metals, whose elastic moduli is in the order of magnitude of metals, are widespread in the biomedical field, especially for bone tissue engineering. The immune reaction and especially the foreign body reaction (FBR) associated with metal implants or prosthetics is drastically lowered due to the spontaneously formed oxide layer at the outmost surface. Accordingly, potentially harmful effects such as fragments resulting from corrosion and ion release in the body are reduced [116]. Common metals in tissue engineering are titanium and its associated alloys such as Nitinol and stainless steel. A large variety of available alloys is defined by the American Society for Testing and Materials (ASTM) and their respective composition is tightly related to their properties [117]. Therefore, “shape memory” properties (i.e., the ability to recover the initial shape after being deformed under thermal variations) can be attributed. This is, for instance, the case for Nitinol [118]. However, metal implants are sensitive to physiological environments as well as to repetitive stresses, which happen in articulations for instance, promoting fatigue and abrasion [119,120]. According to their composition, alloys can slow down the degradation rate of the degradation process and resist against external stresses. Magnesium-based alloys whose residues are eliminated out of the body are a good example for this case [121,122]. Metallic scaffolds can be generated by additive manufacturing technologies [123,124], thereby, three dimensional structures with complex geometries can be achieved as demonstrated by Kolken et al. [125] who produced an auxetic metal-based scaffold (i.e., with a negative value of the Poisson ratio) as a potential hip implant with compression resisting properties. Auxetic materials were already found to be efficient as esophageal stent for patients suffering from dysphagia [126]. Besides, these materials usually have a hierarchically organized structure, which may be suitable for tissue engineering purposes, however, to our knowledge, there is no study in the literature showing the use of auxetic material-based scaffolds *in vitro*.

## 4. Review of the Influence of the Scaffold Architecture on Cell Behavior

Porous three-dimensional environments provide several cues which can affect the ability of cells to sustain their functions. Moreover, the architecture of scaffolds such as the pore and interconnection size or the porosity was considered as an important element influencing mass transport and migration properties, vascularization potential and cellular organization. On the basis of mechanotransduction, external forces are transmitted through actin filaments and translated into biological signals toward the nucleus triggering various signalization pathways. Therefore, the mechanical properties of the based material along with the curved interface provided by the pore walls act as physical cues and thus play a role on cell fate. Below, we summarize the main correlations which have been established between the material structure and its influence on cell growth and fate within the produced scaffolds.

### 4.1. Effect of Porosity

**Figure 4:**
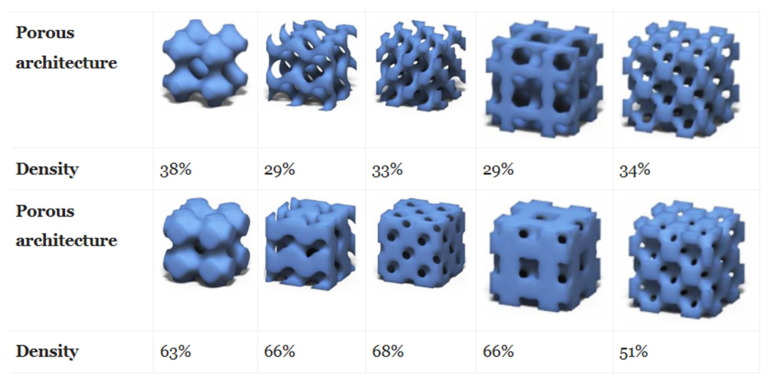
Examples of unit cell design with triply periodic minimal structures (TPMS) with various relative densities ($\rho_{s}/\rho_{b}$) as potential scaffolds for tissue engineering. Adapted with permission from Montazerian et al. [137].

Porosity is an indication of the void percentage of a porous structure and it affects cell growth. Porosity is defined as [127]:

(1) $$P=100\times \left(1-\frac{\rho_{s}}{\rho_{b}}\right),$$

where $\rho_{s}$ and $\rho_{b}$ are the densities of the scaffold and the bulk material, respectively. The Young’s modulus $E_{s}$ of cellular materials is defined as [127]:

(2) $$E_{s}=E_{b}{\left(\frac{\rho_{s}}{\rho_{b}}\right)}^{2},$$

where $E_{b}$ is the Young’s modulus of the bulk material. Hence, from Equations (1) and (2), one can see that an increase in porosity is accompanied with a decrease in Young’s modulus [128,129]. Commonly, generated scaffolds have porosities ranging between 70 and 90% [130,131]. Generally, scaffolds with low porosities have a larger surface area, which is more favorable for initial cell attachment, whereas scaffolds with large porosities, the cell density may be smaller and this delays cell proliferation [132]. On one hand, a higher porosity is correlated with an increase in diffusivity of nutrients and a higher hydraulic permeability, but on the other hand, the loss in Young’s modulus diminishes the mechanical properties of the scaffold [133]. Furthermore, the decrease of the Young’s modulus is higher for biodegradable materials since they lose their integrity during degradation [134,135]. However, this effect can partially be counterbalanced by the newly synthesized ECM formed inside the scaffold, which reinforces the overall mechanical strength of the scaffold as it has been reported earlier for bone [136]. Therefore, porosity value affects both the available surface area and the fluid transport properties which need to be finely balanced to obtain an optimized scaffold. For this purpose, Montazerian et al. [137] demonstrated that triply periodic minimal structures (TPMS) (i.e., a structure whose the sum of the principal curvature at each point is zero [138]) appear as promising candidates for scaffolds with large porosities and transport properties while keeping suitable mechanical properties. Moreover, complex geometries can be achieved, leading to hierarchically organized structures as shown in Figure 4.

### 4.2. Effect of Pore Size and Shape

The diameter of pores in a porous scaffold is the main parameter which can be controlled by most of the used techniques and also probably the most widely studied one. As it has been shown to influence cell fate, in this section we will summarize main conclusions from the literature concerning the effect of pore size on cell behavior. In the below-mentioned studies, even if some exogenous factors were supplemented in some cases, their concentrations and exposure durations were kept constant for all testing conditions to ensure that the differences in cell behavior was only attributed to the varying parameter (i.e., pore size and shape). Matsiko et al. [139] investigated the effect of the pore diameter of a scaffold on MSCs growth by comparing three different diameters in average namely: 94, 130, and 300 µm. Chondrogenic markers were significantly upregulated in scaffolds with the largest pore average diameter (300 µm) according to the secretions of type II collagen (COL2) and sulphated glycosaminoglycans (s-GAGs) after 28 days *in vitro*. These effects were attributed to the better access to nutrients and oxygen in the scaffold with the largest pores even though no quantification of transport properties was made. Besides, cells adopted different morphologies ranging from elongated and flattened in the smallest pores (94 µm) to rounded in scaffolds with the largest pores (300 µm). Conversely, the viability of human skin fibroblasts was improved in scaffolds whose pore diameters in average were either 74 or 160 µm compared to 194 and 381 µm after one week of culture [140]. In another study, scaffolds with a pore diameter of 200 ± 85 µm were found to be optimal regarding tissue growth compared to 170 ± 80 µm, and 243 ± 95 µm-sized pores while the porosity was held fairly constant between 75 and 82% for all the scaffolds [141]. The influence of the thickness of the scaffold was also investigated showing that thicker samples (4 mm) limited cell penetration at the center of the scaffold, which was unlikely for thinner ones (1.5 mm). Tissue like bone has a hierarchically organized structure where pore size and porosity change from cancellous to cortical bone. Di Luca et al. [142] investigated cell growth in scaffolds with a pore diameter gradient ranging from 500 to 1100 µm and showed that ALP activity was higher in the largest pores, which was again justified by a better access to nutrients and oxygen. Interestingly, Heang Oh et al. [143] demonstrated that a pore diameter range of 370 to 400 µm (compared to smaller pores in the range of 90–300 µm) was the most effective range to promote adipose stem cells chondrogenic differentiation using the same argument of nutrient accessibility. Even if in the last two examples, induction medium was added, the pore size effect contribution was distinguished as a key element involved in the differentiation process.

Globally, most of the studies come to agree on pore size ranging between 100 and 700 µm [144]. The major argument relies on the transport properties throughout the whole scaffold which are lowered in scaffolds with smaller pore sizes since there is no flux such as the one of the interstitial fluid or blood stream which delivers nutrients continuously to cells. However, an implanted material will be in contact with many different cell types including immune cells such as macrophages or neutrophils. Macrophages were sensitive to the diameter of the pores in-vivo and pore diameters above 80 µm were shown to favor the pro-inflammatory type M1 macrophage polarization within the scaffold [145]. Hence, this raises the question of the consistency between *in vitro* and *in vivo* studies regarding the optimal range of pore diameter that would guarantee suitable outcomes. Thereby, a screening of several cell types in response to a single scaffold may provide some insight on the overall cell behavior. Some work should be focused mainly on cells arriving at the implant site at the early stages after exposure to the body since the first cells in contact with the implant may recruit and secrete different factors which may subsequently determine and regulate the extend of the foreign body reaction (FBR). Moreover, the influence of pore size on cells is implicitly related to the pore shape [146,147] and sometimes, the porosity (which evolves with pore size according to the fabrication process) as well. This multicomponent dependency makes it harder to isolate and quantify the contribution of a single parameter on cell fate. This is even more difficult when realizing that even if many studies have been held, the scaffold material, and thus the chemistry, the fabrication process, the cell type, and culture conditions differ in each case, making it difficult to accurately compare studies with each other.

### 4.3. Effect of Pore Interconnectivity

**Figure 5:**
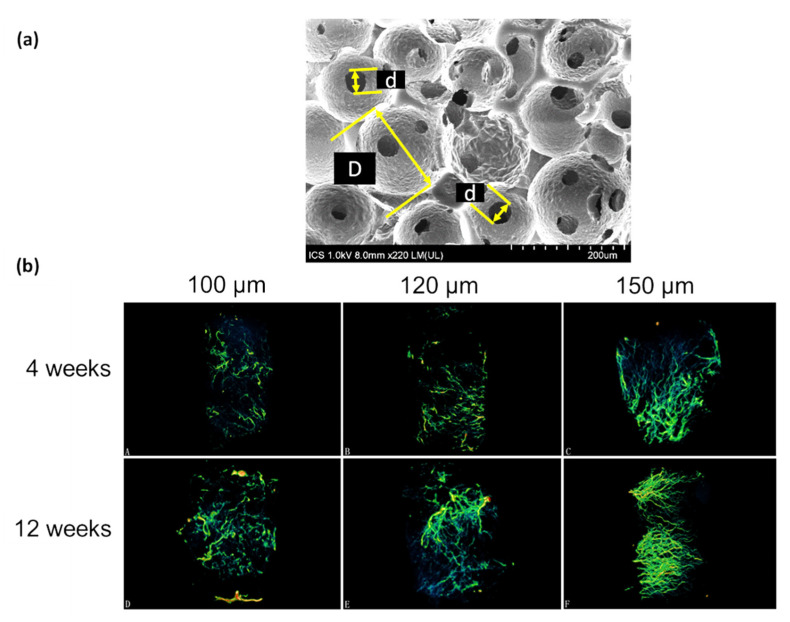
(**a**) SEM image of a porous scaffold produced by sphere templating where “D” indicates the pore diameter and “d” the interconnection diameter (Image taken by G. Lutzweiler). (**b**) Evolution of the blood vessels within a porous scaffold as a function of time with varying interconnection diameters (100, 120, and 150 µm). Adapted from Xiao et al. [149] (Creative Commons CC BY).

The interconnection is the aperture between two adjacent pores (Figure 5a) by which cells must go through in order to fully colonize a scaffold. Despite the fact that this parameter appears to be important regarding cell migration, the relationship between interconnection diameter and cell behavior is poorly documented in the literature compared to pore size and porosity [148]. One reason is that the generation of scaffolds with controlled pore size and interconnectivity remains challenging and is not possible for most of the fabrication processes, although some approaches such as sphere templating and 3D printing allow it [34,149].

In a previous study, sphere templating was employed to produce Beta-tricalcium phosphate (β-TCP) scaffolds where pore diameter was fixed (300–400 µm) and interconnection diameter was varied at 104 ± 13 µm, 117 ± 13 µm and 149 ± 12 µm, respectively [149]. Scaffolds were seeded with human umbilical vein endothelial cells and the results demonstrated that for scaffolds with the largest interconnections (149 µm), the proliferation was the highest along with the PECAM-1 expression (maker for angiogenesis). The same trend was followed *in vivo* regarding the volume of blood vessels formed after 12 weeks in rabbits as shown in Figure 5b.

Somo et al. [40] investigated the influence of two interconnection diameters (33 and 50 µm) for scaffolds having pore sizes ranging between 130 and 150 µm. Changes in the diameter of the interconnections led to modifications of the porosity values: 63% and 77%, respectively. After 3 weeks *in vivo*, scaffolds with the largest interconnections (50 µm) showed more benefits regarding tissue ingrowth, density, and homogeneity of the distribution of blood vessels in rodents. No statistical difference was found after 6 weeks regarding the blood vessel in-growth, which implies that interconnection diameters can enhance the kinetics of the colonization and vascularization of porous scaffolds.

Moreover, Choi et al. [150] produced scaffolds by sphere templating to obtain porous materials with monodisperse or polydisperse pore size distributions. Accordingly, the scaffold with uniform pore diameter leads to a sharper size distribution of the interconnections. They demonstrated that a uniform size distribution of both pores and interconnections resulted in homogeneous cell distribution within the scaffold and to a higher nutrient supply as confirmed by measurements of the hydraulic permeability. The same conclusion was given by Costantini et al. [51] for scaffolds generated by microfluidics.

The hydraulic permeability can be calculated by Darcy’s law, which connects the velocity $V_{D}$ of a fluid flowing through the sample submitted to a pressure gradient providing the driving force of the flow [151].

(3) $$V_{D=-}k\frac{\rho g}{\eta }\frac{\Delta h}{H}.$$

In Equation (3), k is the hydraulic permeability (cm^2^), η is the dynamic viscosity of the fluid (Pa.s), H is the sample thickness (cm), ρ is the density of the fluid (g/cm^3^) g is the gravitational acceleration (9.81 m.s^2^), and Δh is the height difference corresponding to a pressure drop ΔP=ρgΔh.

Many architectural parameters influence the hydraulic permeability such as the interconnection and the pore size but also the number of interconnections per pore named as “interconnectivity” [152]. Thereby, hydraulic permeability is directly linked to the structure of porous scaffolds and consequently, to transport properties of the nutrients and oxygen. Small interconnections lower mass transport and thus lead to a reduced oxygen partial pressure to cells. Interestingly, hypoxia can upregulate the matrix production or even induce differentiation of some cell types such as chondrocytes. Kemppainen et al. [153] showed that scaffolds with small interconnections and subsequently, lower hydraulic permeability can increase the cartilaginous ECM production of chondrocytes.

To conclude this section, cell colonization and vascular growth across porous scaffolds are closely linked to the diameter of the interconnections. The supply of nutrients as well as the removal of cellular wastes are also related to the interconnections and to the interconnectivity by directly affecting the hydraulic permeability. Reduced partial oxygen pressure arising from a lower hydraulic permeability can be advantageous for some cell types such as chondrocytes, which are naturally in a poorly vascularized environment and thus under hypoxic conditions. Cell penetration and cell–cell communication is facilitated, and tissue growth rate is also accelerated in well-interconnected scaffolds.

### 4.4. The Effect of the Curvature

**Figure 6:**
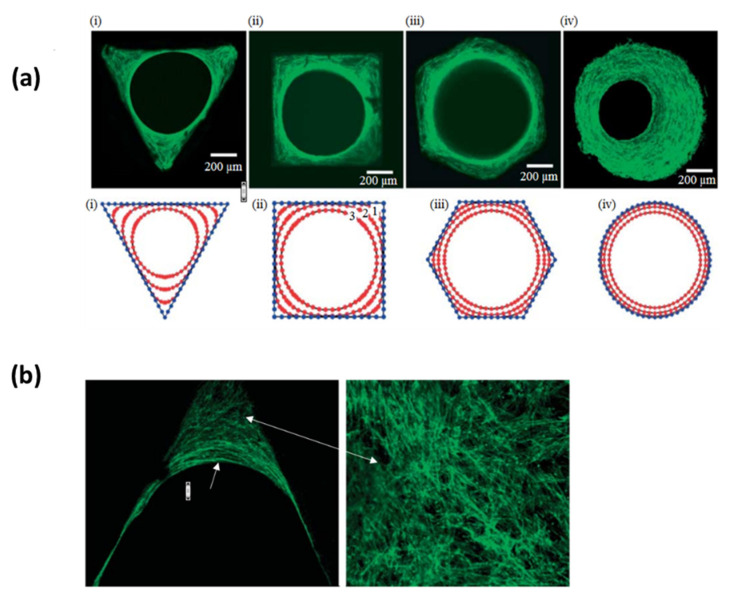
(**a**) Top images show osteoblasts seeded in four different HA plates with various geometries after 21 days (i–iii) and 30 days (iv) to images taken after 21 days of culture and (iv) at 30 days compared with numerical simulations at the bottom. (**b**) F-actin filaments (green) are more disordered in areas with high curvature (close to the edge of the triangle) whereas they are more aligned near the tissue/fluid interface (all images adapted from Rumpler et al. [154], Creative Commons Attribution License).

Cells are sensitive to topographical cues such as roughness but also to the curvature of their substrate. Accordingly, the curved surface provided by porous scaffolds appears as an additional feature which can be involved in the regulation of cell functions. Rumpler et al. [154] demonstrated that the early stages of osteoblasts growth was faster in areas having a high local curvature when cultured in hydroxyapatite (HA) plates with different geometries, either convex, concave of flat surfaces (Figure 6). This curvature-driven tissue growth looks similar to fluids whose surface tension drives the minimization of the interface. Moreover, F-actin filaments showed different organization with respect to the local curvature which was attributed to curvature-induced forces exerted on the cytoskeleton which may subsequently accelerate the tissue grow rate [155]. The formation of blood vessels is also accelerated by concave geometries, which is explained by the aggregation of cells leading to the formation of cell–cell contacts involved in the generation of capillaries [156]. Numerical simulations came at the same conclusion even for scaffolds under flow perfusion [157]. Besides, this phenomenon of curvotaxis can also be used to manipulate the genetic expression of cells [158] and it was shown to even overcome the classical contact guidance exerted by the alignment of ECM fibers on cell orientation [159]. This curvature-driven growth provides a powerful tool to manipulate cell functions without the addition of exogenous factors. Several studies attempted to correlate the tissue formation with a theoretical model which could drive to a predictive manner to anticipate the tissue formation in a given scaffold.

Recently, Buenzli et al. [160] showed that the improved proliferation of cells on concave surfaces is correlated to the local space availability for cells to grow and to the inhomogeneities in cell density. The confinement experienced in concave areas imposes cells to grow along the normal axis to the surface which subsequently participates in the bridging of the pores of scaffolds. Cell migration was also found to be higher in concave areas based on the argument that the confinement imposed on cells is likely to promote protrusion forces arising from the polymerization of actin filaments pushing against the cell membrane [161]. Lastly, the current development of platforms that produce scaffolds with highly complex geometries such as 3D printing may be combined with these predictive models.

### 4.5. Mechanical Properties

The pioneering work conducted by Engler et al. [162] demonstrated how to guide the fate of stem cells by tuning the elasticity of a given substrate. Indeed, cells exert either tensile or pulling forces on their substrate while being also sensitive to forces exerted on them which are transmitted through the cytoskeleton and converted to biological signals [163]. Hence, many studies reported how the influence of changing the Young’s modulus of the substrate is connected to the signal transduction pathway [164,165]. Therefore, the influence of the Young’s modulus of scaffolds was also investigated deeply since mechanotransduction can become an alternative to control the fate of stem cell without the need of soluble molecules such as growth factors. Myoblast cell viability and elongation were higher on scaffold having an E-value of 200 kPa compared to 4, 20, 60, and 280 kPa [166]. Interestingly, Sridharan et al. [167] demonstrated that macrophage polarization depends on the stiffness of collagen scaffold but also on the crosslinking agent. Besides, several parts of the body undergo mechanical stresses naturally which is another mechanical solicitation supported by the cells, hence, several researchers developed systems such as bioreactors in order to mimic such conditions. For instance, fibroblasts seeded into a scaffold undergoing mechanical loadings (uniaxial compression) where shown to upregulate their secretions of procollagen type I, fibronectin and MMPs [168] while MSCs secreted more type I collagen, fibronectin, and lysyl oxidase under cyclic loading [169]. Nonetheless, Wernike et al. [170] demonstrated that cyclic loadings can help chondrocytes seeded into a polyurethane scaffold to maintain their phenotype as assessed by type II collagen measurement for short term culture, despite the fact that hypoxia seemed to be more efficient to prevent chondrocyte de-differentiation. While many studies were focused on the macroscopic level of Young’s modulus (i.e., of the whole material), cells interact only with their surroundings whose local Young’s modulus value can sometimes be smaller than the one of the bulk material [171]. Nonetheless, Grier et al. [172] showed that tenocytes are sensitive to the way that collagen-GAG scaffold resisted to contractile forces of cells. Indeed, tenocytes where able to maintain their phenotype on scaffold with the highest degree of crosslinking (i.e., with the highest stiffness) while cells loss their phenotype on scaffold with a smaller degree of crosslinking. The authors showed that the local mechanical properties could be correlated to the macroscopic Young’s modulus. Therefore, cells are sensitive to their local environment and geometry, especially for cellular materials such as foams, in which cells can exert bending on the pore walls and struts whose local response to an apply stress differs from that of the whole foam [173]. For example, Freyman et al. [174] developed an experimental setup measured the local contractile force exerted by fibroblasts on a collagen-based scaffold. In addition, the role of the matrix viscoelasticity has also become an extensive field of research [72]. Chaudhuri et al. [175] demonstrated that MSCs embedded in an alginate hydrogel coupled with cell-adhesive ligands (RGD), and displaying a fast stress relaxation kinetics allows cells to remodel mechanically the fibers increasing locally the RGD ligand density. Thereby, this high ligand density is involved in the signaling pathway, influencing the lineage commitment of stem cells. Moreover, hydrogels non-covalently crosslinked can improve cell spreading through remodeling of the fibers, displaying a viscoelastic behavior [176].

### 4.6. Bioactive Scaffolds

Despite the evidence that scaffold structure and mechanical properties can guide cellular response, the complete differentiation into a given phenotype is tightly regulated *in vivo* and often requires the use of bioactive components. Moreover, biochemical cues remain predominant over physical cues to control cell fate [177]. Known as the third generation of biomaterials, bioactive scaffolds can be loaded with bioactive agents, functionalized, or even based on natural materials such as ECM components in which some biologically relevant functions are intrinsically present. Macroporous scaffolds can be coupled with bioactive compounds which can be released gradually during the degradation of the material or incorporated into vesicles or micro/nanoparticles where their release profile depends on the degradation kinetics of their respective carrier. For instance, incorporation of Nel-like molecule-1 (Nell-1) growth factors in chitosan nanoparticles loaded in an electrospun scaffold allows to maintain the continuous release of growth factors to cells enhancing in a significant way the type II collagen, *SOX9*, and aggrecan expression of hBMSCs consistent with chondrogenic differentiation [178]. Nanosilicates incorporated in a copolymer based on poly(ethylene oxide terephthalate) (PEOT)/poly(butylene terephthalate) (PBT) (PEOT/PBT) were shown to increase ALP activity of hMSC which indicates an osteogenic differentiation [179]. Furthermore, Sun et al. [180] used a computational approach to demonstrate that the pore size of a scaffold plays a role in the release kinetics from the pore walls of growth factors while porosity affects the vascularization and osteogenesis, which implies that scaffold architecture can contribute to modulate the supply of growth factors. Moreover, cell response can also be stimulated through another functionality of the scaffold: the nanoporosity. Firstly, cells can probe their environment at the nanoscale, asperities of substrates are likely felt as ECM fibers [181] and can guide stem cells toward specific lineages, or conversely, maintain their immunosuppressive phenotype [182]. Many strategies were developed to implement nanocues into 3D scaffolds as the formation of ECM-like nanofibers through electrospinning [183]. Secondly, nanopores contribute to increase the bioactivity of Bioglasses by providing a large specific area, thereby, increasing the dissolution rate of Bioglass, which in turn accelerates the formation of an hydroxyapatite layer involved in bone regeneration [184,185]. The example of bone is particularly relevant here due to its multiscale and multiphasic features and also due to its hierarchically organized porosity [186,187]. Hasan et al. [188] generated a composite scaffold obtained from carboxymethyl cellulose and chitosan loaded with silver nanoparticles as a multifaceted substrate having both regenerative and antimicrobial properties. However, the controlled release of bioactive molecules is still a challenging task considering the complex spatiotemporal regulation of growth factors in tissues [189,190]. Furthermore, complete differentiation of stem cells depends mostly on the combination of several GFs and signals over a certain duration of time (concomitantly or sequentially) [191,192]. Moreover, GFs stability is difficult to control and highly sensitive to the external environment such as temperature or pH [193] and when introduced into the material constituting the scaffold, they can be damaged during the manufacturing process [194]. Shah et al. [192] incorporated both hBMP-2 (recombinant human bone morphogenetic protein-2) and angiogenic rhVEGF165 (recombinant human vascular endothelial growth factor) in a polyelectrolyte multilayer allowing to control simultaneously the doses and the release order mediating the formation of a mature bone. Naturally-derived polymer-based scaffolds are biologically active since proteins naturally contain sequences that can be recognized by cell surface receptors to promote the formation of stable focal adhesions [195] and enzymatically cleavable sequences involved in cell remodeling and migratory processes [196], or specific sites acting as reservoirs to capture and provide soluble molecules to cells [197,198]. For example, self-assembled peptide-based (SAP) hydrogels have a great potential in regenerative medicine since one can generated a highly fibrous assembly which mimics closely the ECM [199]. Nevertheless, SAP-based hydrogels are mostly composed of di-tri peptides due to complex synthesis steps [71], limiting their bioactivity compared to systems based on full-length proteins where several functions are incorporated and localized to participate synergistically in the regulation of cell functions [95]. Supramolecular materials are also one interesting option due to their non-covalent and thus, reversible interactions. Danker et al. [200] developed a ureido-pyrimidinone-based supramolecular material which can be easily functionalized with bioactive peptides allowing fibroblasts to adhere and which was also degradable. It is noteworthy to mention that the cell–material interaction is mostly indirect [201]. Indeed, Loebel et al. [202] showed that hMSCs fate is regulated mostly by their adhesion and remodeling to their own secreted proteins when encapsulated into HA hydrogels implying that the scaffold-mediated cell regulation depends on how secreted ECM components are adsorbed onto the fibers of the hydrogel. One step further in the imitation of the ECM can be achieved by self-healing hydrogels to account for the dynamic and constant remodeling of tissues [203]. This ability to recover its integrity relies mostly on the supramolecular interactions allowing cells to remodel the fibers network while other broken bonds can be reformed allowing to maintain the structural integrity. Additionally, self-healing hydrogels are good candidates for 3D printing technologies unlike classical covalent hydrogels which can be irreversibly damaged during the extrusion process [204]. Finally, other routes are being investigated to develop stimuli responsive hydrogel-based scaffolds, Liu et al. [205] generated a chitosan-based hydrogel with glucose oxidase immobilized on the surface as a promising solution for the regulation of glucose level in blood. pH-sensitive hydrogels are also interesting for drug delivery applications [206].

## 5. *In Vivo* Outcomes and Clinical Trials

Although tissue regeneration assisted by scaffolds led to promising results *in vitro*, the absence of immune system and the whole cascade of events happening at different timelines is difficult to reproduce artificially. Several studies have already been conducted *in vivo*, and the aim of this section is to summarize the main results where the relationship between the scaffold structure and cell response or tissue integration has been explored in order to identify the principal successes and the remaining issues to tackle.

Following the same trend as for *in vitro* studies, the influence of structural parameters like pore and interconnection sizes has also been investigated in animals. Van Tienen et al. [207] produced polyurethane foams having a bi-modal pore size distribution: larger pores with diameters ranging between 150 and 300 µm and smaller pores whose diameters were either in the range of 15–20 µm or above 30 µm. These two types of scaffolds had porosity values of 73 and 86% where the highest value of porosity was found for the scaffold with the largest micropores (>30µm). Results after implantation in rats showed that more tissue was formed in the scaffold with larger micropores. A 6 months study was also conducted in dog meniscus where a polyurethane scaffold was placed. The diameter of the pores was in the 150–355 µm range while the interconnection diameters were below 50 µm. A fibrovascular tissue was formed within the scaffold as a consequence of the FBR confirmed by giant cells at the implant site. The FBR was attributed to the polyurethane–macrophages interaction and results were variable between each animal [207]. Moreover, structures with sharper pore size distributions seem to enhance tissue ingrowth *in vivo* [208]. Furthermore, dendritic cell maturation was found to be higher in scaffolds with pore diameter of 40 µm compared to 90 µm, which led to a collagenous and avascular tissue [209]. Accordingly, the suitable range regarding the pore size seems to follow the same trend *in vivo* and *in vitro*, namely, few hundreds of microns, while positive outcomes regarding immune cell maturation occur preferably for pore size below 100 µm.

The effect of the interconnection diameter was also evaluated. A series of porous β-TCP scaffolds were produced by Feng et al. with pore diameter gradually increasing between 300 and 700 µm with interconnection diameter fixed at 120 µm and constant porosity (70%). After 4 weeks of implantation in rabbits, the authors found that the extend of fibrovascular tissue was inversely proportional to the pore diameter. They argued that smaller diameters (i.e., larger local curvature) accelerate the tissue growth through a curvature-driven mechanism [210]. In previous studies, we demonstrated also that the cellular filling of implant consisting in an assembly of titanium microbeads is facilitated with smaller beads despite these surfaces were convex, cell bridging, and cell–cell contacts were favored [211,212]. Besides, a scaffold with a higher number of interconnections (i.e., the interconnectivity) shows a significant improvement of osteogenesis in rabbits compared to the same scaffold with a limited number of interconnections [213]. This result was attributed to the larger surface area, which provides more available space for cell attachment, while degrading faster which also improves the resorption of the scaffold.

Two main routes are followed in scaffold-based strategies, namely: pre-cellularized scaffolds with autologous cells as a strategy to reduce rejections [214], and more recently, cell-free scaffolds which are under consideration since they are classified as “medical devices” according to the U.S Food and Drug Administration (FDA) standards, thus, their clinical applications could be faster [215]. For instance, bone ingrowth was improved in cell-free porous Ti6Al4V scaffold having a pore size of 300–400 µm after 1 year of implantation in goat bone defect [216]. However, scaffolds seeded with articular chondrocytes were more efficient compared to cell-free scaffolds for meniscus regeneration in rabbits [217].

**Figure 7:**
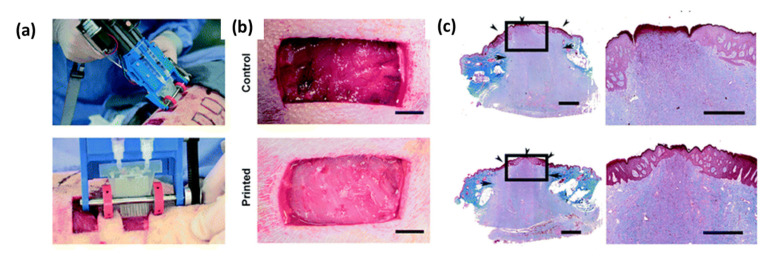
(**a**) Photograph of the printing of a biopolymer sheet in porcine wound. (**b**) Difference between the control (without biopolymer sheet) and the wound filled with a biopolymer sheet after 5 min. Scale bar: 2 mm. (**c**) Re-epithelialization assessed by trichrome staining between control (top images) and wound filled with biopolymer sheet (bottom images), scale bar 2 mm (left), and 1 mm (right). Adapted with permission from Hakimi et al. [218].

Recently, a bioprinter was also designed to print in-situ a sheet composed of biopolymers for wound dressing in porcine model (Figure 7). The printed biomaterial stopped more rapidly bleeding but its regenerative potential was not studied yet [218]. This technology is highly versatile and allows to print either a single or several different layers of various biopolymers, pre-colonized or not, which has a great potential.

Some drug-releasing scaffolds were also tested in animals recently by Fadia et al. [219] which accomplished nerve regeneration in monkeys within a PCL tube coated with microspheres releasing glial cell line–derived neurotrophic factors (GDNF). Very recently, an SAP-based hydrogel, whose chemistry involved a combination of both non-covalent and covalent bonds was generated and allowed for the remodeling and the robustness to stimulate bone regeneration in rabbits. The chosen peptide sequence contributed to high water retention and stimulated the formation of blood vessels. Moreover, the gel was injectable, which avoids any extensive surgery [220].

Clinical trials related to the implantation of scaffolds remain rather rare, and this can be explained by the complex regulatory and by the fact that the development of a functional scaffold is expensive. Furthermore, animal studies cannot necessarily be extrapolated into humans accurately even if they are mandatory [221]. Nonetheless, commercialized scaffolds do already exist. Scaffolds developed for the treatment of foot ulcers based on collagen (Apligraf^®^) or polyglactin (Dermagraft^®^) (Organogenesis. Inc) allowed a successful tissue ingrowth [222,223]. Besides, Integra^®^ (Integra Lifesciences) is a skin substitute based on a top layer composed of the association of a GAG and collagen supported by a silicon layer [224]. Researchers from our lab conceived an implant based on porous titanium for larynx replacement [225]. A 16-month follow-up showed that the patient recovered swallowing and whispering capacities thanks to the artificial larynx.

Furthermore, scaffolds based on polyurethane were developed as a meniscus substitute (Actifit) [226]. After at least five years post-implantation, MRI scans revealed that cartilage remained mostly immature even though functional scores were suitable. However, the pain felt by some patients was too high to continue the study. A collagenous scaffold was implanted in five patients having a spinal cord defect, where two out of five patients showed partial neural regeneration, which was attributed to the favorable environment provided by the scaffold [227]. Furthermore, a polymeric scaffold made by a combination of lactic-co-glycolic acid and poly(L-lysine) prepared by salt leaching process did not induce any complication after 6 months in a patient. The scaffold was implanted at a spinal cord defect and a slight improvement of sensorimotor functions was noticed [228]. Although it is not clearly mentioned, the absence of inflammatory complication could be correlated with the pore size which was shown to prevent the penetration of fibroblasts that are involved in the formation of scar tissues [229]. In another clinical study, bioactive glass having both nanopores and micropores showed that alveolar bone remodeling was improved at the pocket resulting from tooth extraction. The addition of nanopores within the structure contributed to increase the surface area and therefore, to the dissolution whose products participate in the remodeling process [230]. A SAP-based hydrogel named P_11_-4 showed to behave as a nucleation agent in the formation of hydroxyapatite and showed in caries lesion treatments, enamel regeneration in humans [231], this same system also promoted regeneration of rat bone cranial defects after 6 weeks [232].

It turns out that in several studies, tissue in-growth within the porous scaffold is not a limitation *in vivo* conversely to *in vitro* where the lack of interstitial fluid and blood stream together with the absence of chemotactic gradient may limit the migration potential of the cells at the center of the scaffold. However, *in vivo* and especially in clinical trials, the neo tissue is often fibrotic and non-functional potentially leading to implant failure. This deficiency comes from the inflammatory process whose evolution deviates from the normal wound healing cascade due to the presence of the foreign body. Immune cells such as macrophages play a leading role in the healing process. Indeed, macrophage polarization, either M1 which is attributed to a pro inflammatory pathway or M2 known as pro regenerative [233] are key elements influencing the cascade of events after implantation [234]. Scaffolds with an average pore diameter of 40 µm can induce a shift in macrophage polarization from M1 to M2 type leading to a decrease of the FBR associated with a higher vascularization 4 weeks post implantation in rats [145]. This discovery provides the opportunity to modulate macrophage polarization and therefore, the host response to implants at the early stages via pore diameters. Several studies agreed on a limit diameter of the pores of the scaffolds set at 80 µm above which the fibrous capsule is increased and less blood vessels are formed within the scaffolds [39,235]. Sadtler et al. [236] demonstrated that the shift toward M2 lineage depends on the presence of T-helpers 2 cells at the implant site which secreted IL4, which is subsequently involved in the M2 commitment of macrophages.

## 6. General Conclusion and Future Perspectives

In the view of the literature, some general trends and agreements can be extracted regarding the structural parameters of scaffolds. First, pore size from a few hundred microns are mostly defined as the most suitable to guarantee an access to nutrients for cells. Moreover, porosity values are also usually found above 70%, which provides space for cell infiltration and mass transport while providing enough material for initial cell attachment. Interconnection size is also a key element affecting transport properties throughout the porous structure and one acceptable consensus seems to converge around 50 µm as minimum to allow blood vessels formation and cell migration. Physical cues such as the material stiffness, its viscoelasticity, or pore curvature can participate in the regulation of cell differentiation and functions but the effect of endogenous factors such as GFs, hormones or cytokines remains stronger to drive cell fate toward a fully mature state. Nevertheless, the complex regulation of signaling molecules in tissues is hard to reproduce artificially, taking into account the spatio-temporal and multicomponent-dependent supply of bioactive molecules. Platforms allowing a combinatorial provision of both physical and chemical cues are relevant approaches to address this issue [177,237]. This raises the question of the complexity to process scaffolds having various functionalities and being able to evolve and to adapt to the dynamic process of wound healing encountered in biological environments, and accordingly, their associated cost to develop such systems. Moreover, most of the studies performed *in vitro* consist of seeding cells (either stem cells or adult cells) into a porous scaffold and looking at the influence of one or several components on those cells. *In vivo*, immune cells such as neutrophils, monocytes, and later on, macrophages are more likely the first cells that come in contact with the scaffold, therefore, these cells subsequently, adhere, and secrete cytokines to recruit other actors whereas the migration and differentiation of stem cells at the site of implantation corresponds only to the later stages of the healing process. For example, Barthes et al. [238] recently modified the surface of titanium microbeads to diminish macrophage adhesion, while increasing fibroblasts colonization at the same time. Consequently, the state of the scaffold (i.e., the protein type adsorbed onto the surface and the cells already present) may probably be different from the one of the raw scaffolds freshly generated and immersed in culture medium *in vitro*. Such discrepancies were already pointed out in the evaluation of the pore size influence on macrophage polarization and their subsequent influence on the capsule formation. Indeed, pore size below 100 µm appears as preferable. *In vivo* outcomes also support these trends since implanted scaffolds are often filled with scar tissues with low functionality and as a consequence of the foreign body reaction [239]. Furthermore, the immune cascade is also patient-dependent, emphasizing the need to orient future research toward more personalized scaffolds as recently stressed by Vrana et al. [240].

## References

[1] Langer R., Vacanti J.P. (1993). Tissue engineering. Science.

[2] Ratner B.D. (2016). A pore way to heal and regenerate: 21st century thinking on biocompatibility. Regen. Biomater..

[3] Grand View Research Tissue Engineering Market Size, Share & Trends Report Tissue Engineering Market Size, Share & Trends Analysis Report By Application. https://www.grandviewresearch.com/industry-analysis/tissue-engineering-and-regeneration-industry.

[4] Antoni D., Burckel H., Josset E., Noel G. (2015). Three-dimensional cell culture: A breakthrough in vivo. Int. J. Mol. Sci..

[5] Ito Y. (1999). Surface micropatterning to regulate cell functions. Biomaterials.

[6] Lucas B., Pérez L.M., Gálvez B.G. (2018). Importance and regulation of adult stem cell migration. J. Cell. Mol. Med..

[7] Dalby M.J., Gadegaard N., Tare R., Andar A., Riehle M.O., Herzyk P., Wilkinson C.D.W., Oreffo R.O.C. (2007). The control of human mesenchymal cell differentiation using nanoscale symmetry and disorder. Nat. Mater..

[8] Zadpoor A.A. (2015). Bone tissue regeneration: The role of scaffold geometry. Biomater. Sci..

[9] Kishan A.P., Cosgriff-Hernandez E.M. (2017). Recent advancements in electrospinning design for tissue engineering applications: A review. J. Biomed. Mater. Res. Part A.

[10] Haider A., Haider S., Kang I.-K. (2018). A comprehensive review summarizing the effect of electrospinning parameters and potential applications of nanofibers in biomedical and biotechnology. Arab. J. Chem..

[11] Ndreu A., Nikkola L., Ylikauppila H., Ashammakhi N., Hasirci V. (2008). Electrospun biodegradable nanofibrous mats for tissue engineering. Nanomedicine.

[12] Agarwal S., Wendorff J.H., Greiner A. (2008). Use of electrospinning technique for biomedical applications. Polymer.

[13] Müller K., Quinn J.F., Johnston A.P.R., Becker M., Greiner A., Caruso F. (2006). Polyelectrolyte functionalization of electrospun fibers. Chem. Mater..

[14] Kenawy E.-R., Bowlin G.L., Mansfield K., Layman J., Simpson D.G., Sanders E.H., Wnek G.E. (2002). Release of tetracycline hydrochloride from electrospun poly (ethylene-co-vinylacetate), poly (lactic acid), and a blend. J. Control. Release.

[15] Yu D.-G., Shen X.-X., Branford-White C., White K., Zhu L.-M., Bligh S.W.A. (2009). Oral fast-dissolving drug delivery membranes prepared from electrospun polyvinylpyrrolidone ultrafine fibers. Nanotechnology.

[16] Powell H.M., Boyce S.T. (2009). Engineered Human Skin Fabricated Using Electrospun Collagen–PCL Blends: Morphogenesis and Mechanical Properties. Tissue Eng. Part A.

[17] Shields K.J., Beckman M.J., Bowlin G.L., Wayne J.S. (2004). Mechanical Properties and Cellular Proliferation of Electrospun Collagen Type II. Tissue Eng..

[18] Halili A.N., Kürüm B., Karahan S., Hasirci V. (2018). Collagen Based Multilayer Scaffolds for Meniscus Tissue Engineering: In Vivo Test Results. Biomater. Med. Appl..

[19] Stachewicz U., Szewczyk P.K., Kruk A., Barber A.H., Czyrska-Filemonowicz A. (2019). Pore shape and size dependence on cell growth into electrospun fiber scaffolds for tissue engineering: 2D and 3D analyses using SEM and FIB-SEM tomography. Mater. Sci. Eng. C.

[20] Gizaw M., Faglie A., Pieper M., Poudel S., Chou S.-F. (2019). The Role of Electrospun Fiber Scaffolds in Stem Cell Therapy for Skin Tissue Regeneration. Med One.

[21] Kuo T.-Y., Lin C.-M., Hung S.-C., Hsien T.-Y., Wang D.-M., Hsieh H.-J. (2018). Incorporation and selective removal of space-forming nanofibers to enhance the permeability of cytocompatible nanofiber membranes for better cell growth. J. Taiwan Inst. Chem. Eng..

[22] Melchels F.P.W., Domingos M.A.N., Klein T.J., Malda J., Bartolo P.J., Hutmacher D.W. (2012). Additive manufacturing of tissues and organs. Prog. Polym. Sci..

[23] Ovsianikov A., Yoo J., Mironov V. (2018). 3D Printing and Biofabrication.

[24] Lee J.-Y., An J., Chua C.K. (2017). Fundamentals and applications of 3D printing for novel materials. Appl. Mater. Today.

[25] Liu A., Xue G., Sun M., Shao H., Ma C., Gao Q., Gou Z., Yan S., Liu Y., He Y. (2016). 3D printing surgical implants at the clinic: A experimental study on anterior cruciate ligament reconstruction. Sci. Rep..

[26] Yeo M., Lee H., Kim G.H. (2016). Combining a micro/nano-hierarchical scaffold with cell-printing of myoblasts induces cell alignment and differentiation favorable to skeletal muscle tissue regeneration. Biofabrication.

[27] Castilho M., Dias M., Gbureck U., Groll J., Fernandes P., Pires I., Gouveia B., Rodrigues J., Vorndran E. (2013). Fabrication of computationally designed scaffolds by low temperature 3D printing. Biofabrication.

[28] Bose S., Vahabzadeh S., Bandyopadhyay A. (2013). Bone tissue engineering using 3D printing. Mater. Today.

[29] Sun A.X., Lin H., Beck A.M., Kilroy E.J., Tuan R.S. (2015). Projection stereolithographic fabrication of human adipose stem cell-incorporated biodegradable scaffolds for cartilage tissue engineering. Front. Bioeng. Biotechnol..

[30] Cui X., Boland T., Lima D., Lotz M. (2012). Thermal inkjet printing in tissue engineering and regenerative medicine. Recent Pat. Drug Deliv. Formul..

[31] Bella C., Fosang A., Donati D.M., Wallace G.G., Choong P.F.M. (2015). 3D bioprinting of cartilage for orthopedic surgeons: Reading between the lines. Front. Surg..

[32] Mosadegh B., Xiong G., Dunham S., Min J.K. (2015). Current progress in 3D printing for cardiovascular tissue engineering. Biomed. Mater..

[33] Shahriari D., Koffler J.Y., Tuszynski M.H., Campana W.M., Sakamoto J.S. (2017). Hierarchically ordered porous and high-volume polycaprolactone microchannel scaffolds enhanced axon growth in transected spinal cords. Tissue Eng. Part A.

[34] Lutzweiler G., Barthès J., Koenig G., Kerdjoudj H., Mayingi J., Boulmedais F., Schaaf P., Drenckhan W., Vrana N.E. (2019). Modulation of Cellular Colonization of Porous Polyurethane scaffolds via the control of pore interconnection size and nanoscale surface modifications. ACS Appl. Mater. Interfaces.

[35] Zhao K., Tang Y.F., Qin Y.S., Luo D.F. (2011). Polymer template fabrication of porous hydroxyapatite scaffolds with interconnected spherical pores. J. Eur. Ceram. Soc..

[36] Linnes M.P., Ratner B.D., Giachelli C.M. (2007). A fibrinogen-based precision microporous scaffold for tissue engineering. Biomaterials.

[37] Lutzweiler G., Farago J., Oliveira E., Jacomine L., Erverdi O., Vrana N.E., Testouri A., Schaaf P., Drenckhan W. (2020). Validation of Milner’s visco-elastic theory of sintering for the generation of porous polymers with finely tuned morphology. Soft Matter.

[38] Descamps M., Duhoo T., Monchau F., Lu J., Hardouin P., Hornez J.C., Leriche A. (2008). Manufacture of macroporous β-tricalcium phosphate bioceramics. J. Eur. Ceram. Soc..

[39] Sussman E.M., Halpin M.C., Muster J., Moon R.T., Ratner B.D. (2014). Porous Implants Modulate Healing and Induce Shifts in Local Macrophage Polarization in the Foreign Body Reaction. Ann. Biomed. Eng..

[40] Somo S.I., Akar B., Bayrak E.S., Larson J.C., Appel A.A., Mehdizadeh H., Cinar A., Brey E.M. (2015). Pore Interconnectivity Influences Growth Factor-Mediated Vascularization in Sphere-Templated Hydrogels. Tissue Eng. Part C Methods.

[41] Silbert L.E. (2010). Jamming of frictional spheres and random loose packing. Soft Matter.

[42] Jacobs L.J.M., Kemmere M.F., Keurentjes J.T.F. (2008). Sustainable polymer foaming using high pressure carbon dioxide: A review on fundamentals, processes and applications. Green Chem..

[43] Costantini M., Barbetta A., Deng Y., Kuiper J.B.T. (2018). 6-Gas foaming technologies for 3D scaffold engineering. Functional 3D Tissue Engineering Scaffolds: Materials, Technologies, and Applications.

[44] Deb P., Deoghare A.B., Borah A., Barua E., Lala S. (2017). Das Scaffold Development Using Biomaterials: A Review. Mater. Today.

[45] Dehli F., Rebers L., Stubenrauch C., Southan A. (2019). Highly ordered gelatin methacryloyl hydrogel foams with tunable pore size. Biomacromolecules.

[46] Drenckhan W., Saint-Jalmes A. (2015). The science of foaming. Adv. Colloid Interface Sci..

[47] Isabelle C., Sylvie C.A., Florence E., Francois G., Reinhard H., Olivier P., Florence R., Flatman S.C. (2013). Foams: Structure and Dynamics.

[48] Testouri A., Ranft M., Honorez C., Kaabeche N., Ferbitz J., Freidank D., Drenckhan W. (2013). Generation of Crystalline Polyurethane Foams Using Millifluidic Lab-on-a-Chip Technologies. Adv. Eng. Mater..

[49] Andrieux S., Drenckhan W., Stubenrauch C. (2017). Highly ordered biobased scaffolds: From liquid to solid foams. Polymer.

[50] Andrieux S., Quell A., Stubenrauch C., Drenckhan W. (2018). Liquid foam templating--A route to tailor-made polymer foams. Adv. Colloid Interface Sci..

[51] Costantini M., Colosi C., Mozetic P., Jaroszewicz J., Tosato A., Rainer A., Trombetta M., Więszkowski W., Dentini M., Barbetta A. (2016). Correlation between porous texture and cell seeding efficiency of gas foaming and microfluidic foaming scaffolds. Mater. Sci. Eng. C.

[52] Costantini M., Colosi C., Jaroszewicz J., Tosato A., Więszkowski W., Dentini M., Garstecki P., Barbetta A. (2015). Microfluidic Foaming: A Powerful Tool for Tailoring the Morphological and Permeability Properties of Sponge-like Biopolymeric Scaffolds. ACS Appl. Mater. Interfaces.

[53] Rossmy G.R., Kollmeier H.J., Lidy W., Schator H., Wiemann M. (1977). Cell-opening in one-shot flexible polyether based polyurethane foams. The Role of Silicone Surfactant and its Foundation in the Chemistry of Foam Formation. J. Cell. Plast..

[54] Yasunaga K., Neff R.A., Zhang X.D., Macosko C.W. (1996). Study of Cell Opening in Flexible Polyurethane Foam. J. Cell. Plast..

[55] Li H., Liu K., Sang Q., Williams G.R., Wu J., Wang H., Wu J., Zhu L.-M. (2017). A thermosensitive drug delivery system prepared by blend electrospinning. Colloids Surfaces B Biointerfaces.

[56] Lutzweiler G. (2019). Matériaux Poreux à Base de Polyuréthane Pour L’ingénierie Tissulaire.

[57] Ng W.S., Lee C.S., Chuah C.H., Cheng S.-F. (2017). Preparation and modification of water-blown porous biodegradable polyurethane foams with palm oil-based polyester polyol. Ind. Crops Prod..

[58] Janik H., Marzec M. (2015). A review: Fabrication of porous polyurethane scaffolds. Mater. Sci. Eng. C.

[59] Choi S.-W., Zhang Y., MacEwan M.R., Xia Y. (2013). Neovascularization in biodegradable inverse opal scaffolds with uniform and precisely controlled pore sizes. Adv. Healthc. Mater..

[60] Chiessi E., Cavalieri F., Paradossi G. (2007). Water and polymer dynamics in chemically cross-linked hydrogels of poly (vinyl alcohol): A molecular dynamics simulation study. J. Phys. Chem. B.

[61] Kang W., Bi B., Zhuo R., Jiang X. (2017). Photocrosslinked methacrylated carboxymethyl chitin hydrogels with tunable degradation and mechanical behavior. Carbohydr. Polym..

[62] Kumar P., Ciftci S., Barthes J., Knopf-Marques H., Muller C.B., Debry C., Vrana N.E., Ghaemmaghami A.M. (2020). A composite Gelatin/hyaluronic acid hydrogel as an ECM mimic for developing mesenchymal stem cell-derived epithelial tissue patches. J. Tissue Eng. Regen. Med..

[63] Satish L., Millan S., Das S., Jena S., Sahoo H. (2017). Thermal aggregation of bovine serum albumin in conventional buffers: An insight into molecular level interactions. J. Solut. Chem..

[64] Andersen T., Auk-Emblem P., Dornish M. (2015). 3D cell culture in alginate hydrogels. Microarrays.

[65] Yang K.-S., Guo X., Meng W., Hyun J.-Y., Kang I.-K., Kim Y. (2003). Behavior of hepatocytes inoculated in gelatin-immobilized polyurethane foam. Macromol. Res..

[66] Schaschkow A., Sigrist S., Mura C., Barthes J., Vrana N.E., Czuba E., Lemaire F., Neidl R., Dissaux C., Lejay A. (2020). Glycaemic control in diabetic rats treated with islet transplantation using plasma combined with hydroxypropylmethyl cellulose hydrogel. Acta Biomater..

[67] Almany L., Seliktar D. (2005). Biosynthetic hydrogel scaffolds made from fibrinogen and polyethylene glycol for 3D cell cultures. Biomaterials.

[68] Chen Z., Zhao D., Liu B., Nian G., Li X., Yin J., Qu S., Yang W. (2019). 3D Printing of Multifunctional Hydrogels. Adv. Funct. Mater..

[69] Lee C.R., Grad S., Gorna K., Gogolewski S., Goessl A., Alini M. (2005). Fibrin--polyurethane composites for articular cartilage tissue engineering: A preliminary analysis. Tissue Eng..

[70] Criado-Gonzalez M., Fores J.R., Wagner D., Schröder A.P., Carvalho A., Schmutz M., Harth E., Schaaf P., Jierry L., Boulmedais F. (2019). Enzyme-assisted self-assembly within a hydrogel induced by peptide diffusion. Chem. Commun..

[71] Frederix P.W.J.M., Scott G.G., Abul-Haija Y.M., Kalafatovic D., Pappas C.G., Javid N., Hunt N.T., Ulijn R.V., Tuttle T. (2015). Exploring the sequence space for (tri-) peptide self-assembly to design and discover new hydrogels. Nat. Chem..

[72] Chaudhuri O. (2017). Viscoelastic hydrogels for 3D cell culture. Biomater. Sci..

[73] Qin Z., Yu X., Wu H., Li J., Lv H., Yang X. (2019). Nonswellable and Tough Supramolecular Hydrogel Based on Strong Micelle Cross-Linkings. Biomacromolecules.

[74] Alakpa E.V., Jayawarna V., Lampel A., Burgess K.V., West C.C., Bakker S.C.J., Roy S., Javid N., Fleming S., Lamprou D.A. (2016). Tunable supramolecular hydrogels for selection of lineage-guiding metabolites in stem cell cultures. Chem.

[75] Jeon O., Bouhadir K.H., Mansour J.M., Alsberg E. (2009). Photocrosslinked alginate hydrogels with tunable biodegradation rates and mechanical properties. Biomaterials.

[76] Szot C.S., Buchanan C.F., Freeman J.W., Rylander M.N. (2011). 3D in vitro bioengineered tumors based on collagen I hydrogels. Biomaterials.

[77] Ling Y., Rubin J., Deng Y., Huang C., Demirci U., Karp J.M., Khademhosseini A. (2007). A cell-laden microfluidic hydrogel. Lab Chip.

[78] Bryant S.J., Cuy J.L., Hauch K.D., Ratner B.D. (2007). Photo-patterning of porous hydrogels for tissue engineering. Biomaterials.

[79] Kostina N.Y., Blanquer S., Pop-Georgievski O., Rahimi K., Dittrich B., Höcherl A., Michálek J., Grijpma D.W., Rodriguez-Emmenegger C. (2019). Zwitterionic Functionalizable Scaffolds with Gyroid Pore Architecture for Tissue Engineering. Macromol. Biosci..

[80] Ahearne M., Yang Y., Liu K.K. (2008). Mechanical characterisation of hydrogels for tissue engineering applications. Top. Tissue Eng..

[81] Murphy S.V., De Coppi P., Atala A. (2019). Opportunities and challenges of translational 3D bioprinting. Nat. Biomed. Eng..

[82] Foster G.A., Headen D.M., González C.G., Salmerón-Sánchez M., Shirwan H., Garc A.J. (2017). Protease-degradable microgels for protein delivery for vascularization. Biomaterials.

[83] Karg M., Pich A., Hellweg T., Hoare T., Lyon L.A., Crassous J.J., Suzuki D., Gumerov R.A., Schneider S., Potemkin I.I. (2019). Nanogels and microgels: From model colloids to applications, recent developments, and future trends. Langmuir.

[84] Rubinstein M., Colby R.H. (2003). Polymer Physics.

[85] Manavitehrani I., Fathi A., Badr H., Daly S., Negahi Shirazi A., Dehghani F. (2016). Biomedical Applications of Biodegradable Polyesters. Polymers.

[86] Castilla-Cortázar I., Más-Estellés J., Meseguer-Dueñas J.M., Escobar Ivirico J.L., Marí B., Vidaurre A. (2012). Hydrolytic and enzymatic degradation of a poly(ε-caprolactone) network. Polym. Degrad. Stab..

[87] Kannan R.Y., Salacinski H.J., Butler P.E., Hamilton G., Seifalian A.M. (2005). Current status of prosthetic bypass grafts: A review. J. Biomed. Mater. Res. Part B Appl. Biomater..

[88] Winnacker M. (2017). Polyamides and their functionalization: Recent concepts for their applications as biomaterials. Biomater. Sci..

[89] Lutzweiler G., Barthes J., Vrana N.E., Rawiso M., Louis B., Mayingi J., Carre A., Drenckhan W., Schaaf P. (2020). Adjustment of Cell Adhesion on Polyurethane Structures via Control of the Hard/Soft Segment Ratio. Macromol. Mater. Eng..

[90] Adipurnama I., Yang M.-C., Ciach T., Butruk-Raszeja B. (2017). Surface modification and endothelialization of polyurethane for vascular tissue engineering applications: A review. Biomater. Sci..

[91] Ma Z., Mao Z., Gao C. (2007). Surface modification and property analysis of biomedical polymers used for tissue engineering. Colloids Surfaces B Biointerfaces.

[92] Williams R. (2010). Surface Modification of Biomaterials: Methods Analysis and Applications.

[93] Mason T.O., Shimanovich U. (2018). Fibrous Protein Self-Assembly in Biomimetic Materials. Adv. Mater..

[94] Neves S.C., Pereira R.F., Araújo M., Barrias C.C., Barbosa M.A., Martins M.C. (2018). 4-Bioengineered peptide-functionalized hydrogels for tissue regeneration and repair. Peptides and Proteins as Biomaterials for Tissue Regeneration and Repair.

[95] Trujillo S., Gonzalez-Garcia C., Rico P., Reid A., Windmill J., Dalby M.J., Salmeron-Sanchez M. (2019). Engineered full-length Fibronectin-based hydrogels sequester and present growth factors to promote regenerative responses in vitro and in vivo. bioRxiv.

[96] Dinoro J., Maher M., Talebian S., Jafarkhani M., Mehrali M., Orive G., Foroughi J., Lord M.S., Dolatshahi-Pirouz A. (2019). Sulfated polysaccharide-based scaffolds for orthopaedic tissue engineering. Biomaterials.

[97] Malafaya P.B., Silva G.A., Reis R.L. (2007). Natural–origin polymers as carriers and scaffolds for biomolecules and cell delivery in tissue engineering applications. Adv. Drug Deliv. Rev..

[98] Lee C.H., Singla A., Lee Y. (2001). Biomedical applications of collagen. Int. J. Pharm..

[99] Caló E., Khutoryanskiy V.V. (2015). Biomedical applications of hydrogels: A review of patents and commercial products. Eur. Polym. J..

[100] Gerecht S., Burdick J.A., Ferreira L.S., Townsend S.A., Langer R., Vunjak-Novakovic G. (2007). Hyaluronic acid hydrogel for controlled self-renewal and differentiation of human embryonic stem cells. Proc. Natl. Acad. Sci. USA.

[101] Elisseeff J., Ferran A., Hwang S., Varghese S., Zhang Z. (2006). The Role of Biomaterials in Stem Cell Differentiation: Applications in the Musculoskeletal System. Stem Cells Dev..

[102] Sabir M.I., Xu X., Li L. (2009). A review on biodegradable polymeric materials for bone tissue engineering applications. J. Mater. Sci..

[103] Lehn J.-M. (1988). Supramolecular Chemistry—Scope and Perspectives Molecules, Supermolecules, and Molecular Devices (Nobel Lecture). Angew. Chem. Int. Ed. Engl..

[104] Webber M.J., Appel E.A., Meijer E.W., Langer R. (2015). Supramolecular biomaterials. Nat. Mater..

[105] Kisiday J., Jin M., Kurz B., Hung H., Semino C., Zhang S., Grodzinsky A.J. (2002). Self-assembling peptide hydrogel fosters chondrocyte extracellular matrix production and cell division: Implications for cartilage tissue repair. Proc. Natl. Acad. Sci. USA.

[106] Ellis-Behnke R.G., Liang Y.-X., You S.-W., Tay D.K.C., Zhang S., So K.-F., Schneider G.E. (2006). Nano neuro knitting: Peptide nanofiber scaffold for brain repair and axon regeneration with functional return of vision. Proc. Natl. Acad. Sci. USA.

[107] Naskar J., Palui G., Banerjee A. (2009). Tetrapeptide-Based Hydrogels: For Encapsulation and Slow Release of an Anticancer Drug at Physiological pH. J. Phys. Chem. B.

[108] Kutz M. (2003). Others Standard Handbook of Biomedical Engineering and Design.

[109] Thomas S., Balakrishnan P., Sreekala M.S. (2018). Fundamental Biomaterials: Ceramics.

[110] Rezwan K., Chen Q.Z., Blaker J.J., Boccaccini A.R. (2006). Biodegradable and bioactive porous polymer/inorganic composite scaffolds for bone tissue engineering. Biomaterials.

[111] Ducheyne P., Mauck R.L., Smith D.H. (2012). Biomaterials in the repair of sports injuries. Nat. Mater..

[112] Hench L.L. (2006). The story of Bioglass^®^. J. Mater. Sci. Mater. Med..

[113] De Bruijn J.D., Bovell Y.P., Davies J.E., van Blitterswijk C.A. (1994). Osteoclastic resorption of calcium phosphates is potentiated in postosteogenic culture conditions. J. Biomed. Mater. Res..

[114] Doi Y., Shibutani T., Moriwaki Y., Kajimoto T., Iwayama Y. (1998). Sintered carbonate apatites as bioresorbable bone substitutes. J. Biomed. Mater. Res..

[115] Paramsothy M., Ramakrishna S. (2015). Biodegradable Materials for Clinical Applications: A Review. Rev. Adv. Sci. Eng..

[116] Gotman I. (1997). Characteristics of metals used in implants. J. Endourol..

[117] Welsch G., Boyer R., Collings E.W. (1993). Materials Properties Handbook: Titanium Alloys.

[118] Duerig T., Pelton A., Stöckel D. (1999). An overview of nitinol medical applications. Mater. Sci. Eng. A.

[119] Kunčická L., Kocich R., Lowe T.C. (2017). Advances in metals and alloys for joint replacement. Prog. Mater. Sci..

[120] Liu X., Chu P.K., Ding C. (2004). Surface modification of titanium, titanium alloys, and related materials for biomedical applications. Mater. Sci. Eng. R Rep..

[121] Yang K., Zhou C., Fan H., Fan Y., Jiang Q., Song P., Fan H., Chen Y., Zhang X. (2018). Bio-Functional Design, Application and Trends in Metallic Biomaterials. Int. J. Mol. Sci..

[122] Zheng Y.F., Gu X.N., Witte F. (2014). Biodegradable metals. Mater. Sci. Eng. R Rep..

[123] Brandt M. (2016). Laser Additive Manufacturing: Materials, Design, Technologies, and Applications.

[124] Murr L.E., Gaytan S.M., Ramirez D.A., Martinez E., Hernandez J., Amato K.N., Shindo P.W., Medina F.R., Wicker R.B. (2012). Metal fabrication by additive manufacturing using laser and electron beam melting technologies. J. Mater. Sci. Technol..

[125] Kolken H.M.A., Janbaz S., Leeflang S.M.A., Lietaert K., Weinans H.H., Zadpoor A.A. (2018). Rationally designed meta-implants: A combination of auxetic and conventional meta-biomaterials. Mater. Horizons.

[126] Ali M.N., Busfield J.J.C., Rehman I.U. (2014). Auxetic oesophageal stents: Structure and mechanical properties. J. Mater. Sci. Mater. Med..

[127] Gibson L.J., Ashby M.F. (1999). Cellular Solids: Structure and Properties.

[128] Haiying Y., Howard W.M., Paul H.W., Shang-you Y. (2008). Effect of Porosity and Pore Size on Microstructures and Mechanical Properties of Poly-e-Caprolactone-Hydroxyapatite Composites. J. Biomed. Mater. Res. Part B Appl. Biomater..

[129] Sobral J.M., Caridade S.G., Sousa R.A., Mano J.F., Reis R.L. (2011). Three-dimensional plotted scaffolds with controlled pore size gradients: Effect of scaffold geometry on mechanical performance and cell seeding efficiency. Acta Biomater..

[130] Shimko D.A., Shimko V.F., Sander E.A., Dickson K.F., Nauman E.A. (2005). Effect of porosity on the fluid flow characteristics and mechanical properties of tantalum scaffolds. J. Biomed. Mater. Res. Part B Appl. Biomater..

[131] Danilevicius P., Georgiadi L., Pateman C.J., Claeyssens F., Chatzinikolaidou M., Farsari M. (2015). The effect of porosity on cell ingrowth into accurately defined, laser-made, polylactide-based 3D scaffolds. Appl. Surf. Sci..

[132] Torres-Sanchez C., Al Mushref F.R.A., Norrito M., Yendall K., Liu Y., Conway P.P. (2017). The effect of pore size and porosity on mechanical properties and biological response of porous titanium scaffolds. Mater. Sci. Eng. C.

[133] Wang Y.F., Barrera C.M., Dauer E.A., Gu W., Andreopoulos F., Huang C.Y.C. (2017). Systematic characterization of porosity and mass transport and mechanical properties of porous polyurethane scaffolds. J. Mech. Behav. Biomed. Mater..

[134] Ikeda R., Fujioka H., Nagura I., Kokubu T., Toyokawa N., Inui A., Makino T., Kaneko H., Doita M., Kurosaka M. (2009). The effect of porosity and mechanical property of a synthetic polymer scaffold on repair of osteochondral defects. Int. Orthop..

[135] Zhang Q., Jiang Y., Zhang Y., Ye Z., Tan W., Lang M. (2013). Effect of porosity on long-term degradation of poly ($\varepsilon$-caprolactone) scaffolds and their cellular response. Polym. Degrad. Stab..

[136] Murphy W.L., Kohn D.H., Mooney D.J. (2000). Growth of continuous bonelike mineral within porous poly (lactide-co-glycolide) scaffolds in vitro. J. Biomed. Mater. Res..

[137] Montazerian H., Davoodi E., Asadi-Eydivand M., Kadkhodapour J., Solati-Hashjin M. (2017). Porous scaffold internal architecture design based on minimal surfaces: A compromise between permeability and elastic properties. Mater. Des..

[138] Bobbert F.S.L., Lietaert K., Eftekhari A.A., Pouran B., Ahmadi S.M., Weinans H., Zadpoor A.A. (2017). Additively manufactured metallic porous biomaterials based on minimal surfaces: A unique combination of topological, mechanical, and mass transport properties. Acta Biomater..

[139] Matsiko A., Gleeson J.P., O’Brien F.J. (2014). Scaffold Mean Pore Size Influences Mesenchymal Stem Cell Chondrogenic Differentiation and Matrix Deposition. Tissue Eng. Part A.

[140] Yang J., Shi G., Bei J., Wang S., Cao Y., Shang Q., Yang G., Wang W. (2002). Fabrication and surface modification of macroporous poly (L-lactic acid) and poly (L-lactic-co-glycolic acid)(70/30) cell scaffolds for human skin fibroblast cell culture. J. Biomed. Mater. Res..

[141] Wang H., Pieper J., Péters F., van Blitterswijk C.A., Lamme E.N. (2005). Synthetic scaffold morphology controls human dermal connective tissue formation. J. Biomed. Mater. Res. Part A.

[142] Di Luca A., Ostrowska B., Lorenzo-Moldero I., Lepedda A., Swieszkowski W., Van Blitterswijk C., Moroni L. (2016). Gradients in pore size enhance the osteogenic differentiation of human mesenchymal stromal cells in three-dimensional scaffolds. Sci. Rep..

[143] Oh S.H., Kim T.H., Im G.I., Lee J.H. (2010). Investigation of pore size effect on chondrogenic differentiation of adipose stem cells using a pore size gradient scaffold. Biomacromolecules.

[144] Bružauskait I., Bironait D., Bagdonas E., Bernotien E. (2016). Scaffolds and cells for tissue regeneration: Different scaffold pore sizes—different cell effects. Cytotechnology.

[145] Madden L.R., Mortisen D.J., Sussman E.M., Dupras S.K., Fugate J.A., Cuy J.L., Hauch K.D., Laflamme M.A., Murry C.E., Ratner B.D. (2010). Proangiogenic scaffolds as functional templates for cardiac tissue engineering. Proc. Natl. Acad. Sci. USA.

[146] Rüdrich U., Lasgorceix M., Champion E., Pascaud-Mathieu P., Damia C., Chartier T., Brie J., Magnaudeix A. (2019). Pre-osteoblast cell colonization of porous silicon substituted hydroxyapatite bioceramics: Influence of microporosity and macropore design. Mater. Sci. Eng. C.

[147] Jones A.C., Arns C.H., Hutmacher D.W., Milthorpe B.K., Sheppard A.P., Knackstedt M.A. (2009). The correlation of pore morphology, interconnectivity and physical properties of 3D ceramic scaffolds with bone ingrowth. Biomaterials.

[148] Karande T.S., Ong J.L., Agrawal C.M. (2004). Diffusion in musculoskeletal tissue engineering scaffolds: Design issues related to porosity, permeability, architecture, and nutrient mixing. Ann. Biomed. Eng..

[149] Xiao X., Wang W., Liu D., Zhang H., Gao P., Geng L., Yuan Y., Lu J., Wang Z. (2015). The promotion of angiogenesis induced by three-dimensional porous beta-tricalcium phosphate scaffold with different interconnection sizes via activation of PI3K/Akt pathways. Sci. Rep..

[150] Choi S.-W., Zhang Y., Xia Y. (2010). Three-dimensional scaffolds for tissue engineering: The importance of uniformity in pore size and structure. Langmuir.

[151] Marshall A.J., Ratner B.D. (2005). Quantitative characterization of sphere-templated porous biomaterials. AIChE J..

[152] Murphy W.L., Dennis R.G., Kileny J.L., Mooney D.J. (2002). Salt fusion: An approach to improve pore interconnectivity within tissue engineering scaffolds. Tissue Eng..

[153] Kemppainen J.M., Hollister S.J. (2010). Differential effects of designed scaffold permeability on chondrogenesis by chondrocytes and bone marrow stromal cells. Biomaterials.

[154] Rumpler M., Woesz A., Dunlop J.W.C., van Dongen J.T., Fratzl P. (2008). The effect of geometry on three-dimensional tissue growth. J. R. Soc. Interface.

[155] Bidan C.M., Kommareddy K.P., Rumpler M., Kollmannsberger P., Fratzl P., Dunlop J.W.C. (2013). Geometry as a Factor for Tissue Growth: Towards Shape Optimization of Tissue Engineering Scaffolds. Adv. Healthc. Mater..

[156] Magnaudeix A., Usseglio J., Lasgorceix M., Lalloue F., Damia C., Brie J., Pascaud-Mathieu P., Champion E. (2016). Quantitative analysis of vascular colonisation and angio-conduction in porous silicon-substituted hydroxyapatite with various pore shapes in a chick chorioallantoic membrane (CAM) model. Acta Biomater..

[157] Guyot Y., Papantoniou I., Chai Y.C., Van Bael S., Schrooten J., Geris L. (2014). A computational model for cell/ECM growth on 3D surfaces using the level set method: A bone tissue engineering case study. Biomech. Model. Mechanobiol..

[158] Pieuchot L., Marteau J., Guignandon A., Dos Santos T., Brigaud I., Chauvy P.-F., Cloatre T., Ponche A., Petithory T., Rougerie P. (2018). Curvotaxis directs cell migration through cell-scale curvature landscapes. Nat. Commun..

[159] Werner M., Kurniawan N.A., Korus G., Bouten C.V.C., Petersen A. (2018). Mesoscale substrate curvature overrules nanoscale contact guidance to direct bone marrow stromal cell migration. J. R. Soc. Interface.

[160] Buenzli P.R., Lanaro M., Wong C.S., McLaughlin M.P., Allenby M.C., Woodruff M.A., Simpson M.J. (2020). Cell proliferation and migration explain pore bridging dynamics in 3D printed scaffolds of different pore size. bioRxiv.

[161] He X., Jiang Y. (2017). Substrate curvature regulates cell migration. Phys. Biol..

[162] Engler A.J., Sen S., Sweeney H.L., Discher D.E. (2006). Matrix Elasticity Directs Stem Cell Lineage Specification. Cell.

[163] Humphrey J.D., Dufresne E.R., Schwartz M.A. (2014). Mechanotransduction and extracellular matrix homeostasis. Nat. Rev. Mol. Cell Biol..

[164] Pek Y.S., Wan A.C.A., Ying J.Y. (2010). The effect of matrix stiffness on mesenchymal stem cell differentiation in a 3D thixotropic gel. Biomaterials.

[165] Lozoya O.A., Wauthier E., Turner R.A., Barbier C., Prestwich G.D., Guilak F., Superfine R., Lubkin S.R., Reid L.M. (2011). Regulation of hepatic stem/progenitor phenotype by microenvironment stiffness in hydrogel models of the human liver stem cell niche. Biomaterials.

[166] Levy-Mishali M., Zoldan J., Levenberg S. (2009). Effect of scaffold stiffness on myoblast differentiation. Tissue Eng. Part A.

[167] Sridharan R., Ryan E.J., Kearney C.J., Kelly D.J., O’Brien F.J. (2018). Macrophage polarization in response to collagen scaffold stiffness is dependent on cross-linking agent used to modulate the stiffness. ACS Biomater. Sci. Eng..

[168] Petersen A., Joly P., Bergmann C., Korus G., Duda G.N. (2012). The Impact of Substrate Stiffness and Mechanical Loading on Fibroblast-Induced Scaffold Remodeling. Tissue Eng. Part A.

[169] Baker B.M., Shah R.P., Huang A.H., Mauck R.L. (2011). Dynamic Tensile Loading Improves the Functional Properties of Mesenchymal Stem Cell-Laden Nanofiber-Based Fibrocartilage. Tissue Eng. Part A.

[170] Wernike E., Li Z., Alini M., Grad S. (2008). Effect of reduced oxygen tension and long-term mechanical stimulation on chondrocyte-polymer constructs. Cell Tissue Res..

[171] Roether J., Bertels S., Oelschlaeger C., Bastmeyer M., Willenbacher N. (2018). Microstructure, local viscoelasticity and cell culture suitability of 3D hybrid HA/collagen scaffolds. PLoS ONE.

[172] Grier W.K., Iyoha E.M., Harley B.A.C. (2017). The influence of pore size and stiffness on tenocyte bioactivity and transcriptomic stability in collagen-GAG scaffolds. J. Mech. Behav. Biomed. Mater..

[173] Harley B.A., Freyman T.M., Wong M.Q., Gibson L.J. (2007). A new technique for calculating individual dermal fibroblast contractile forces generated within collagen-GAG scaffolds. Biophys. J..

[174] Freyman T.M., Yannas I.V., Yokoo R., Gibson L.J. (2001). Fibroblast contraction of a collagen--GAG matrix. Biomaterials.

[175] Chaudhuri O., Gu L., Klumpers D., Darnell M., Bencherif S.A., Weaver J.C., Huebsch N., Lee H., Lippens E., Duda G.N. (2015). Hydrogels with tunable stress relaxation regulate stem cell fate and activity. Nat. Mater..

[176] McKinnon D.D., Domaille D.W., Cha J.N., Anseth K.S. (2014). Biophysically Defined and Cytocompatible Covalently Adaptable Networks as Viscoelastic 3D Cell Culture Systems. Adv. Mater..

[177] Ranga A., Gobaa S., Okawa Y., Mosiewicz K., Negro A., Lutolf M.P. (2014). 3D niche microarrays for systems-level analyses of cell fate. Nat. Commun..

[178] Wang C., Hou W., Guo X., Li J., Hu T., Qiu M., Liu S., Mo X., Liu X. (2017). Two-phase electrospinning to incorporate growth factors loaded chitosan nanoparticles into electrospun fibrous scaffolds for bioactivity retention and cartilage regeneration. Mater. Sci. Eng. C.

[179] Carrow J.K., Di Luca A., Dolatshahi-Pirouz A., Moroni L., Gaharwar A.K. (2018). 3D-printed bioactive scaffolds from nanosilicates and PEOT/PBT for bone tissue engineering. Regen. Biomater..

[180] Sun X., Kang Y., Bao J., Zhang Y., Yang Y., Zhou X. (2013). Modeling vascularized bone regeneration within a porous biodegradable CaP scaffold loaded with growth factors. Biomaterials.

[181] Elsdale T., Bard J. (1972). Collagen substrata for studies on cell behavior. J. Cell Biol..

[182] Ross E., Turner L.-A., Saeed A., Burgess K., Blackburn G., Reynolds P., Wells J., Mountford J., Gadegaard N., Salmeron-Sanchez M. (2019). Nanotopography reveals metabolites that maintain the immunosuppressive phenotype of mesenchymal stem cells. bioRxiv.

[183] Park S., Kim D., Park S., Kim S., Lee D., Kim W., Kim J. (2018). Nanopatterned scaffolds for neural tissue engineering and regenerative medicine. Cutting-Edge Enabling Technologies for Regenerative Medicine.

[184] Sepulveda P., Jones J.R., Hench L.L. (2002). In vitro dissolution of melt-derived 45S5 and sol-gel derived 58S bioactive glasses. J. Biomed. Mater. Res..

[185] Jones J.R. (2009). New trends in bioactive scaffolds: The importance of nanostructure. J. Eur. Ceram. Soc..

[186] Zhang K., Wang S., Zhou C., Cheng L., Gao X., Xie X., Sun J., Wang H., Weir M.D., Reynolds M.A. (2018). Advanced smart biomaterials and constructs for hard tissue engineering and regeneration. Bone Res..

[187] Zhu L., Luo D., Liu Y. (2020). Effect of the nano/microscale structure of biomaterial scaffolds on bone regeneration. Int. J. Oral Sci..

[188] Hasan A., Waibhaw G., Saxena V., Pandey L.M. (2018). Nano-biocomposite scaffolds of chitosan, carboxymethyl cellulose and silver nanoparticle modified cellulose nanowhiskers for bone tissue engineering applications. Int. J. Biol. Macromol..

[189] Benedetto A., Accetta G., Fujita Y., Charras G. (2014). Spatiotemporal control of gene expression using microfluidics. Lab Chip.

[190] Perrier-Groult E., Ronzière M.-C., Bareille R., Pinzano A., Mallein-Gerin F., Freyria A.-M. (2011). Analysis of collagen expression during chondrogenic induction of human bone marrow mesenchymal stem cells. Biotechnol. Lett..

[191] Patel Z.S., Young S., Tabata Y., Jansen J.A., Wong M.E.K., Mikos A.G. (2008). Dual delivery of an angiogenic and an osteogenic growth factor for bone regeneration in a critical size defect model. Bone.

[192] Shah N.J., Macdonald M.L., Beben Y.M., Padera R.F., Samuel R.E., Hammond P.T. (2011). Tunable dual growth factor delivery from polyelectrolyte multilayer films. Biomaterials.

[193] Mitchell A.C., Briquez P.S., Hubbell J.A., Cochran J.R. (2016). Engineering growth factors for regenerative medicine applications. Acta Biomater..

[194] Koons G.L., Mikos A.G. (2019). Progress in three-dimensional printing with growth factors. J. Control. release.

[195] D’Souza S.E., Ginsberg M.H., Plow E.F. (1991). Arginyl-glycyl-aspartic acid (RGD): A cell adhesion motif. Trends Biochem. Sci..

[196] Rodriguez-Cabello J.C., De Torre I.G., Ibañez-Fonseca A., Alonso M. (2018). Bioactive scaffolds based on elastin-like materials for wound healing. Adv. Drug Deliv. Rev..

[197] Kang Y.-H., Jeon S.H., Park J.-Y., Chung J.-H., Choung Y.-H., Choung H.-W., Kim E.-S., Choung P.-H. (2011). Platelet-rich fibrin is a Bioscaffold and reservoir of growth factors for tissue regeneration. Tissue Eng. Part A.

[198] Naves A.F., Motay M., Mérindol R., Davi C.P., Felix O., Catalani L.H., Decher G. (2016). Layer-by-Layer assembled growth factor reservoirs for steering the response of 3T3-cells. Colloids Surfaces B Biointerfaces.

[199] Harper M.M., Connolly M.L., Goldie L., Irvine E.J., Shaw J.E., Jayawarna V., Richardson S.M., Dalby M.J., Lightbody D., Ulijn R., Nilsson B.L., Doran T.M. (2018). V Biogelx: Cell Culture on Self-Assembling Peptide Gels BT-Peptide Self-Assembly: Methods and Protocols. Peptide Self-Assembly: Methods and Protocols.

[200] Dankers P.Y.W., Harmsen M.C., Brouwer L.A., Van Luyn M.J.A., Meijer E.W. (2005). A modular and supramolecular approach to bioactive scaffolds for tissue engineering. Nat. Mater..

[201] Ferreira S.A., Motwani M.S., Faull P.A., Seymour A.J., Yu T.T.L., Enayati M., Taheem D.K., Salzlechner C., Haghighi T., Kania E.M. (2018). Bi-directional cell-pericellular matrix interactions direct stem cell fate. Nat. Commun..

[202] Loebel C., Mauck R.L., Burdick J.A. (2019). Local nascent protein deposition and remodelling guide mesenchymal stromal cell mechanosensing and fate in three-dimensional hydrogels. Nat. Mater..

[203] Saunders L., Ma P.X. (2019). Self-Healing Supramolecular Hydrogels for Tissue Engineering Applications. Macromol. Biosci..

[204] Azevedo S., Costa A.M.S., Andersen A., Choi I.S., Birkedal H., Mano J.F. (2017). Bioinspired Ultratough Hydrogel with Fast Recovery, Self-Healing, Injectability and Cytocompatibility. Adv. Mater..

[205] Liu J., Xiao Y., Wang X., Huang L., Chen Y., Bao C. (2019). Glucose-sensitive delivery of metronidazole by using a photo-crosslinked chitosan hydrogel film to inhibit Porphyromonas gingivalis proliferation. Int. J. Biol. Macromol..

[206] Bacelar A.H., Cengiz I.F., Silva-Correia J., Sousa R.A., Oliveira J.M., Reis R.L. (2017). “Smart” hydrogels in tissue engineering and regenerative medicine applications. Handbook of Intelligent Scaffolds for Tissue Engineering and Regenerative Medicine.

[207] Tienen T.G., Heijkants R.G.J.C., de Groot J.H., Pennings A.J., Schouten A.J., Veth R.P.H., Buma P. (2006). Replacement of the knee meniscus by a porous polymer implant: A study in dogs. Am. J. Sports Med..

[208] Kim H.Y., Jung S.Y., Lee S.J., Lee H.J., Truong M.-D., Kim H.S. (2019). Fabrication and characterization of 3D-printed elastic auricular scaffolds: A pilot study. Laryngoscope.

[209] Chen R., Ma H., Zhang L., Bryers J.D. (2018). Precision-porous templated scaffolds of varying pore size drive dendritic cell activation. Biotechnol. Bioeng..

[210] Feng B., Jinkang Z., Zhen W., Jianxi L., Jiang C., Jian L., Guolin M., Xin D. (2011). The effect of pore size on tissue ingrowth and neovascularization in porous bioceramics of controlled architecture in vivo. Biomed. Mater..

[211] Rieger E., Dupret-Bories A., Salou L., Metz-Boutigue M.-H., Layrolle P., Debry C., Lavalle P., Engin Vrana N. (2015). Controlled implant/soft tissue interaction by nanoscale surface modifications of 3D porous titanium implants. Nanoscale.

[212] Vrana N.E., Dupret-Bories A., Schultz P., Debry C., Vautier D., Lavalle P. (2013). Titanium Microbead-Based Porous Implants: Bead Size Controls Cell Response and Host Integration. Adv. Healthc. Mater..

[213] Lu T., Feng S., He F., Ye J. (2020). Enhanced osteogenesis of honeycomb β-tricalcium phosphate scaffold by construction of interconnected pore structure: An in vivo study. J. Biomed. Mater. Res. Part A.

[214] Webber M.J., Khan O.F., Sydlik S.A., Tang B.C., Langer R. (2015). A Perspective on the Clinical Translation of Scaffolds for Tissue Engineering. Ann. Biomed. Eng..

[215] Kon E., Roffi A., Filardo G., Tesei G., Marcacci M. (2015). Scaffold-based cartilage treatments: With or without cells? A systematic review of preclinical and clinical evidence. Arthrosc. J. Arthrosc. Relat. Surg..

[216] Li G., Wang L., Pan W., Yang F., Jiang W., Wu X., Kong X., Dai K., Hao Y. (2016). In vitro and in vivo study of additive manufactured porous Ti6Al4V scaffolds for repairing bone defects. Sci. Rep..

[217] Moradi L., Vasei M., Dehghan M.M., Majidi M., Farzad Mohajeri S., Bonakdar S. (2017). Regeneration of meniscus tissue using adipose mesenchymal stem cells-chondrocytes co-culture on a hybrid scaffold: In vivo study. Biomaterials.

[218] Hakimi N., Cheng R., Leng L., Sotoudehfar M., Ba P.Q., Bakhtyar N., Amini-Nik S., Jeschke M.G., Günther A. (2018). Handheld skin printer: In situ formation of planar biomaterials and tissues. Lab Chip.

[219] Fadia N.B., Bliley J.M., DiBernardo G.A., Crammond D.J., Schilling B.K., Sivak W.N., Spiess A.M., Washington K.M., Waldner M., Liao H.-T. (2020). Long-gap peripheral nerve repair through sustained release of a neurotrophic factor in nonhuman primates. Sci. Transl. Med..

[220] Okesola B.O., Ni S., Derkus B., Galeano C.C., Hasan A., Wu Y., Ramis J., Buttery L., Dawson J.I., D’Este M. (2020). Growth-Factor Free Multicomponent Nanocomposite Hydrogels That Stimulate Bone Formation. Adv. Funct. Mater..

[221] Madry H., Alini M., Stoddart M.J., Evans C., Miclau T., Steiner S. (2014). Barriers and strategies for the clinical translation of advanced orthopaedic tissue engineering protocols. Eur. Cell Mater..

[222] Naughton G., Mansbridge J., Gentzkow G. (1997). A Metabolically Active Human Dermal Replacement for the Treatment of Diabetic Foot Ulcers. Artif. Organs.

[223] Falanga V., Sabolinski M. (1999). A bilayered living skin construct (APLIGRAF^®^) accelerates complete closure of hard-to-heal venous ulcers. Wound Repair Regen..

[224] Machens H.-G., Berger A.C., Mailaender P. (2000). Bioartificial skin. Cells Tissues Organs.

[225] Debry C., Vrana N.E., Dupret-Bories A. (2017). Implantation of an Artificial Larynx after Total Laryngectomy. N. Engl. J. Med..

[226] Leroy A., Beaufils P., Faivre B., Steltzlen C., Boisrenoult P., Pujol N. (2017). Actifit^®^ polyurethane meniscal scaffold: MRI and functional outcomes after a minimum follow-up of 5 years. Orthop. Traumatol. Surg. Res..

[227] Xiao Z., Tang F., Tang J., Yang H., Zhao Y., Chen B., Han S., Wang N., Li X., Cheng S. (2016). One-year clinical study of NeuroRegen scaffold implantation following scar resection in complete chronic spinal cord injury patients. Sci. China Life Sci..

[228] Theodore N., Hlubek R., Danielson J., Neff K., Vaickus L., Ulich T.R., Ropper A.E. (2016). First human implantation of a bioresorbable polymer scaffold for acute traumatic spinal cord injury: A clinical pilot study for safety and feasibility. Neurosurgery.

[229] Teng Y.D., Lavik E.B., Qu X., Park K.I., Ourednik J., Zurakowski D., Langer R., Snyder E.Y. (2002). Functional recovery following traumatic spinal cord injury mediated by a unique polymer scaffold seeded with neural stem cells. Proc. Natl. Acad. Sci. USA.

[230] El Shazley N., Hamdy A., El-Eneen H.A., El Backly R.M., Saad M.M., Essam W., Moussa H., El Tantawi M., Jain H., Marei M.K. (2016). Bioglass in alveolar bone regeneration in orthodontic patients: Randomized controlled clinical trial. JDR Clin. Transl. Res..

[231] Brunton P.A., Davies R.P.W., Burke J.L., Smith A., Aggeli A., Brookes S.J., Kirkham J. (2013). Treatment of early caries lesions using biomimetic self-assembling peptides--a clinical safety trial. Br. Dent. J..

[232] Saha S., Yang X.B., Wijayathunga N., Harris S., Feichtinger G.A., Davies R.P.W., Kirkham J. (2019). A biomimetic self-assembling peptide promotes bone regeneration in vivo: A rat cranial defect study. Bone.

[233] Alvarez M.M., Liu J.C., Trujillo-de Santiago G., Cha B.H., Vishwakarma A., Ghaemmaghami A.M., Khademhosseini A. (2015). Delivery strategies to control inflammatory response: Modulating M1-M2 polarization in tissue engineering applications. J. Control. Release.

[234] Velnar T., Bailey T., Smrkolj V. (2009). The Wound Healing Process: An Overview of the Cellular and Molecular Mechanisms. J. Int. Med. Res..

[235] Marshall A.J. (2004). Biomaterials with tightly controlled poresize that promote vascular in-growth. Polym. Prepr..

[236] Sadtler K., Estrellas K., Allen B.W., Wolf M.T., Fan H., Tam A.J., Patel C.H., Luber B.S., Wang H., Wagner K.R. (2016). Developing a pro-regenerative biomaterial scaffold microenvironment requires T helper 2 cells. Science.

[237] Hook A.L., Anderson D.G., Langer R., Williams P., Davies M.C., Alexander M.R. (2010). High throughput methods applied in biomaterial development and discovery. Biomaterials.

[238] Barthes J., Cazzola M., Muller C., Dollinger C., Debry C., Ferraris S., Spriano S., Vrana N.E. (2020). Controlling porous titanium/soft tissue interactions with an innovative surface chemical treatment: Responses of macrophages and fibroblasts. Mater. Sci. Eng. C.

[239] Anderson J.M., Rodriguez A., Chang D.T. (2008). Foreign body reaction to biomaterials. Semin. Immunol..

[240] Vrana N.E., Ghaemmaghami A.M., Zorlutuna P. (2019). Adverse Reactions to Biomaterials: State of the Art in Biomaterial Risk Assessment, Immunomodulation and in vitro Models for Biomaterial Testing. Front. Bioeng. Biotechnol..

